# Cytoskeleton stiffness regulates cellular senescence and innate immune response in Hutchinson–Gilford Progeria Syndrome

**DOI:** 10.1111/acel.13152

**Published:** 2020-07-25

**Authors:** Xiaodong Mu, Chieh Tseng, William S. Hambright, Polina Matre, Chih‐Yi Lin, Palas Chanda, Wanqun Chen, Jianhua Gu, Sudheer Ravuri, Yan Cui, Ling Zhong, John P. Cooke, Laura J. Niedernhofer, Paul D. Robbins, Johnny Huard

**Affiliations:** ^1^ Department of Molecular Physiology and Biophysics Baylor College of Medicine Houston Texas; ^2^ Department of Orthopaedic Surgery McGovern Medical School University of Texas Health Science Center at Houston Houston Texas; ^3^ Shandong First Medical University & Shandong Academy of Medical Sciences Ji'nan China; ^4^ Center for Regenerative Sports Medicine Steadman Philippon Research Institute Vail Colorado; ^5^ Department of Cardiovascular Sciences Houston Methodist Research Institute Houston Texas; ^6^ Electron Microscopy Core Houston Methodist Research Institute Houston Texas; ^7^ Institute on the Biology of Aging and Metabolism and Department of Biochemistry, Molecular Biology and Biophysics University of Minnesota Minneapolis Minnesota

**Keywords:** accelerated aging, cell nucleus, cellular senescence, skeletal muscle, stem cells

## Abstract

Hutchinson–Gilford progeria syndrome (HGPS) is caused by the accumulation of mutant prelamin A (progerin) in the nuclear lamina, resulting in increased nuclear stiffness and abnormal nuclear architecture. Nuclear mechanics are tightly coupled to cytoskeletal mechanics via lamin A/C. However, the role of cytoskeletal/nuclear mechanical properties in mediating cellular senescence and the relationship between cytoskeletal stiffness, nuclear abnormalities, and senescent phenotypes remain largely unknown. Here, using muscle‐derived mesenchymal stromal/stem cells (MSCs) from the *Zmpste24* ^−/−^ (*Z24* ^−/−^) mouse (a model for HGPS) and human HGPS fibroblasts, we investigated the mechanical mechanism of progerin‐induced cellular senescence, involving the role and interaction of mechanical sensors RhoA and Sun1/2 in regulating F‐actin cytoskeleton stiffness, nuclear blebbing, micronuclei formation, and the innate immune response. We observed that increased cytoskeletal stiffness and RhoA activation in progeria cells were directly coupled with increased nuclear blebbing, Sun2 expression, and micronuclei‐induced cGAS‐Sting activation, part of the innate immune response. Expression of constitutively active RhoA promoted, while the inhibition of RhoA/ROCK reduced cytoskeletal stiffness, Sun2 expression, the innate immune response, and cellular senescence. Silencing of Sun2 expression by siRNA also repressed RhoA activation, cytoskeletal stiffness and cellular senescence. Treatment of *Zmpste24^−^* ^/^ *^−^* mice with a RhoA inhibitor repressed cellular senescence and improved muscle regeneration. These results reveal novel mechanical roles and correlation of cytoskeletal/nuclear stiffness, RhoA, Sun2, and the innate immune response in promoting aging and cellular senescence in HGPS progeria.

## Graphical abstract

There is increased RhoA activation and cytoskeleton stiffness in progeria cells, which mediates increased nuclear blebbing, micronuclei formation, innate immune response, and cellular senescence. RhoA activation is coupled with elevated Sun2 expression, but not Sun1, especially in nucleus with blebbing and micronuclei. Repression of RhoA and Sun2 activation was able to reduce cytoskeleton stiffness, nuclear blebbing, and micronuclei formation and delay the progression of cellular senescence.

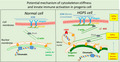

## 1 INTRODUCTION

Hutchinson–Gilford progeria syndrome (HGPS) is a rare, fatal genetic disorder caused by the mutation of *LMNA* (lamin A) gene (Schreiber & Kennedy, 2013). As a nucleoskeletal protein at nuclear lamina, lamin A is essential for mechanical support of the nucleus and is required for the structural link between the nucleoskeleton and cytoskeleton (Phillip, Aifuwa, Walston, & Wirtz, 2015). *LMNA* gene mutation in HGPS leads to the production of mutant prelamin A (progerin), which accumulates at the nuclear envelope (NE) and causes dramatic changes in the nuclear architecture, including thickening of the nuclear lamina, increased nuclear stiffness and nuclear irregularity (nuclear blebbing, a hallmark of HGPS cells), and impaired nucleus deformation capacity (Cao et al., 2011; Dahl et al., 2006; Phillip et al., 2015; Verstraeten, Ji, Cummings, Lee, & Lammerding, 2008; Young, Fong, & Michaelis, 2005). This abnormal nuclear architecture confers additional adverse cellular processes, such as disruption of chromatin anchoring on the laminar structure, inappropriate reorganization of chromatin, telomere dislocation, dysfunction and erosion, delayed response in DNA damage repair and thereby increased DNA damage, and accelerated senescence (Cao et al., 2011; Phillip et al., 2015). Progerin accumulation can also cause natural age‐associated increase in nuclear stiffness, abnormal histone modification patterns, global changes in gene expression, and impaired cell function (Cao et al., 2011; Pacheco et al., 2014; Phillip et al., 2015). In contrast to HGPS cells, lamin A knockout (*LMNA* ^−/−^) cells do not express lamin A and were reported to develop decreased mechanical *stiffness* in both the nucleus/nucleoskeleton and cytoskeleton (Broers et al., 2004; Kim et al., 2017; Schreiber & Kennedy, 2013). Although the increased nuclear stiffness in HGPS cells had been previously observed (Booth, Spagnol, Alcoser, & Dahl, 2015; Dahl et al., 2006), changes in cytoskeletal stiffness in progerin‐expressing cells and the potential correlation of cytoskeletal stiffness with nuclear abnormalities and progeria phenotypes have not been examined.

The cell nucleus is tightly integrated into the structural network of the cytoplasm through linker of the nucleoskeleton and cytoskeleton (LINC) complexes, which contain Sun1/2 proteins (Isermann & Lammerding, 2013; Phillip et al., 2015). Lamin A/C is required for this structural connection of nucleus and cytoskeleton (Isermann & Lammerding, 2013; Phillip et al., 2015), which is essential for a broad range of cellular functions. Mechanical stimuli to the cells can be transmitted from the extracellular matrix (ECM) to the nucleus via the cytoskeleton (Isermann & Lammerding, 2013; Phillip et al., 2015). The mechanical properties of the ECM, especially the stiffness of the local environment, can have a direct and profound impact on the expression of mechano‐responsive genes and the organization of cytoskeletal and nucleoskeletal proteins (Isermann & Lammerding, 2013; Phillip et al., 2015).

Filamentous actin (F‐actin), a key component of the cytoskeleton, is a major determinant of a cell's mechanical properties and is critical to stress response. F‐actin plays a crucial role in mediating ECM‐nuclear mechanical coupling. The stiffness of local environment influences cellular functions via regulating cytoskeletal structure, and cells on stiffer substrates exhibited increased F‐actin polymerization and stress fiber formation with an order of orientation. Lamin A/C is connected to F‐actin cytoskeleton via Sun proteins (Sun1/2), which can mediate the formation of the perinuclear apical actin cap to regulate the nuclear structural integrity (Hoffman et al., 2020; Kim et al., 2017; Lei et al., 2009). Normal actin cytoskeletal dynamics can protect cells from stress by modulating the stress response and cell death (Baird et al., 2014; Gourlay, Carpp, Timpson, Winder, & Ayscough, 2004). Actin cytoskeletal dynamics is closely regulated by the activity of Rho GTPases, particularly RhoA (Sit & Manser, 2011). RhoA is crucial for regulating cell morphology, migration, adhesion, autophagosome formation and function, and many more events associated with F‐actin dynamics (Li et al., 2011). The interaction of RhoA with key mechano‐sensing factors located at the cell membrane, cytoplasm/cytoskeleton, or nuclear membrane has been well documented (Li et al., 2011). In particular, our recent study demonstrated the co‐activation of RhoA with pro‐inflammatory, pro‐fibrogenic, and pro‐osteogenic signaling factors in skeletal muscles of muscular dystrophic mice (Mu et al., 2013).

Moreover, RhoA also plays a role in determining the commitment of stem cell fate, by regulating cytoskeletal mechanics and interacting with mechanical signaling pathways involved in stem cell differentiation (Li et al., 2011). Cell shape, mechanical cues, and cytoskeletal tension were found to regulate the switch in lineage commitment of MSCs by modulating RhoA activity (Li et al., 2011; McBeath, Pirone, Nelson, Bhadriraju, & Chen, 2004). Abnormal RhoA regulation can lead to the dysregulated adipo‐osteogenic balance, which has been linked to various pathological conditions, such as aging, obesity, osteopenia, osteopetrosis, and osteoporosis (Li et al., 2011). Indeed, dysregulated adipo‐osteogenic balance also has been reported in HGPS MSCs with increased osteogenic, but decreased adipogenic potential (Meshorer & Gruenbaum, 2008). Thus, it is possible that RhoA signaling may play a role in conferring the dysregulated adipo‐osteogenic balance in HGPS cells.

Based on these previous findings, we hypothesized that there is increased cytoskeletal stiffness and RhoA activation in HGPS cells, possibly as a response to increased nuclear stiffness, DNA damage, and/or production of reactive oxygen species (ROS). We also hypothesized that increased cytoskeletal stiffness and RhoA activation are associated with accelerated cellular senescence. Thus, we examined cytoskeletal stiffness and RhoA activation in cells from HGPS patients and from a mouse model of HGPS due to a deficiency in Zmpste24 (Z24), a zinc metalloproteinase involved in the formation of mature lamin A. *Z24* ^−/−^ mice have premature onset of aging‐related musculoskeletal changes, similar to those observed in HGPS (Bergo et al., 2002; Fong et al., 2004; Yang et al., 2006). Through a gain and loss of RhoA function, we examined whether changes in cytoskeletal stiffness and RhoA activation in progeria cells correlate with nuclear blebbing, Sun1/2 expression, and micronuclei‐induced cGAS‐Sting activation, part of the innate immune response that has also been linked to senescence. Because the LINC complex containing Sun2 was found to promote focal adhesion assembly by activating RhoA (Thakar, May, Rogers, & Carroll, 2017), we also examined the effect of siRNA‐mediated Sun2 inhibition on cytoskeleton stiffness, perinuclear actin cap formation, nuclear blebbing, and cellular senescence.

## 2 RESULTS

### 2.1 ***MSCs from muscle of Z24*** ^−/−^ ***mice have elevated expression of senescence‐associated secretory phenotype (SASP) factors that negatively impacts muscle stem cell function***

**Figure 1:**
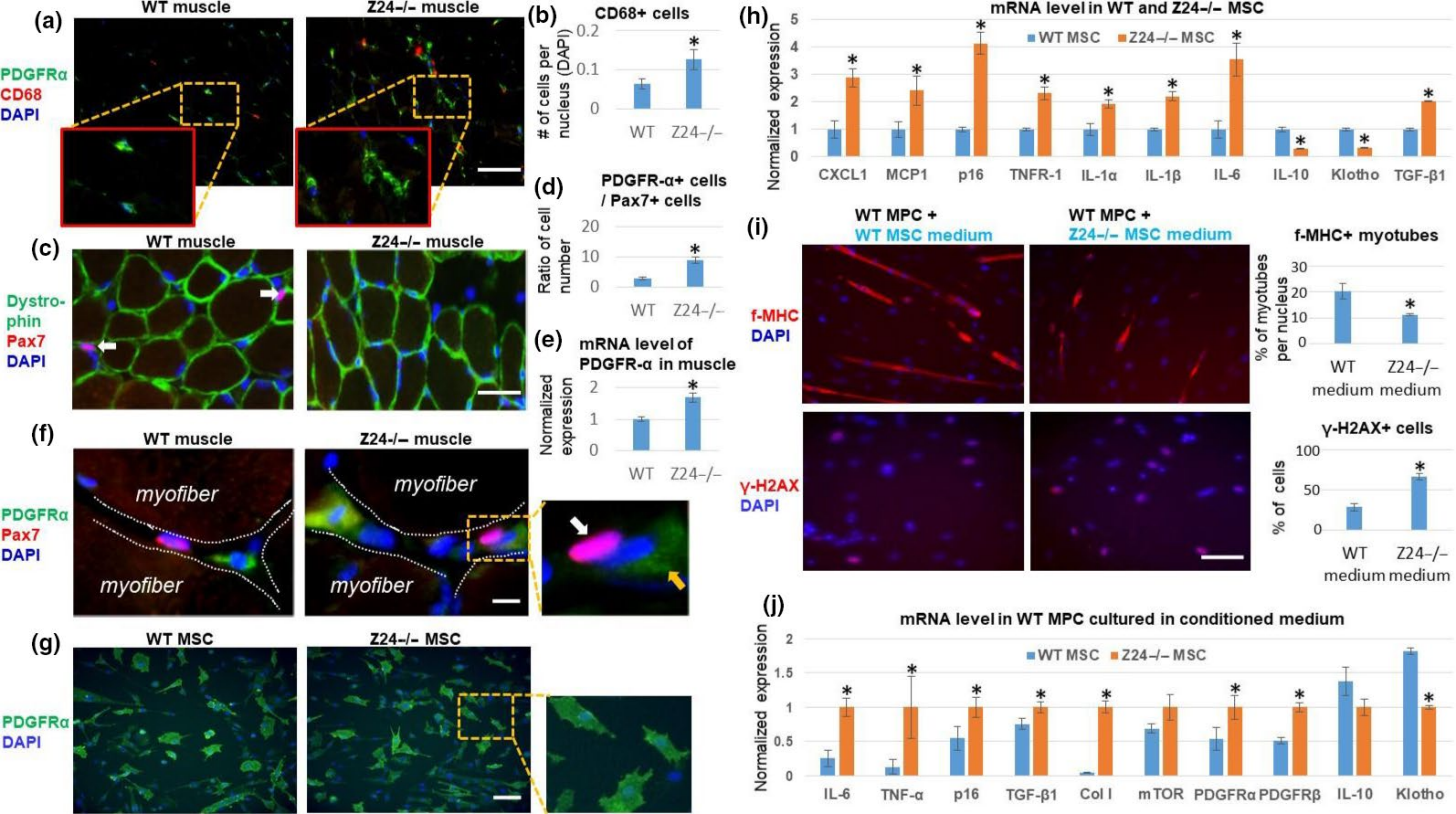
Increased expression of SASP factors in *Z24* ^−/−^ MSCs negatively impacts muscle stem cell function. (a) Gastrocnemius (GM) skeletal muscle tissues were harvested from 5‐month‐old *Z24* ^−/−^ and wild‐type (WT) mice. Immunostaining analysis of PDGFR‐α and CD68 showed increased number of PDGFR‐α^+^ MSCs and CD68^+^ macrophages. Scale bar = 100 µm. (b) Quantification of CD68^+^ cells is shown. (c) Immunostaining analysis of dystrophin and Pax7 showed a decreased number of Pax7^+^ muscle stem cell in *Z24* ^−/−^ mice. (d) Quantification of the ratio of the PDGFR‐α^+^ cells to Pax7^+^ cells is shown. (d) Immunostaining analysis of PDGFR‐α and Pax7 demonstrating a close interaction between these two types of cells in stem cell niche. White arrow indicates a Pax7^+^ cell; orange arrow indicates a PDGFR‐α^+^ cell. Scale bar = 50 µm. (e) Quantification of mRNA level of PDGFR‐α in muscles is shown. (f) Immunostaining analysis of PDGFR‐α and Pax7 to show their relative localization at stem cell niche. Scale bar = 10µm. (g) Immunostaining analysis of PDGFR‐α in mesenchymal stem/stromal cells (MSCs) from WT mice and *Z24* ^−/−^ mice. Scale bar = 100 µm. (h) qPCR results of mRNA from WT MSC and *Z24* ^−/−^ MSCs. (i) Treatment of WT muscle progenitor cells (MPCs) with conditioned medium from WT or *Z24* ^−/−^ MSCs to check the impact on myogenesis potential [the formation of fast‐myosin heavy chain (f‐MHC)‐positive myotubes], and level of DNA damage (γ‐H2AX). Quantitation of cells positive with f‐MHC or γ‐H2AX is shown. Scale bar = 100 µm. (j) qPCR results of mRNA from WT MPCs treated with conditioned medium from WT or *Z24* ^−/−^ MSCs. Data are shown as mean ± standard error. *N *≥ 6. * indicates *p* < .05

Mesenchymal stromal/stem cells in skeletal muscle are nonmyogenic and express platelet‐derived growth factor receptors (PDGFRs). These PDGFR‐α^+^ cells often function as fibro‐adipogenic progenitors (FAPs) and have been found as the main cell type that contributes to pathological fibrosis and fat infiltration in diseased and injured muscles (Contreras, Rebolledo, Oyarzun, Olguin, & Brandan, 2016). Analysis of skeletal muscle from *Z24* ^−/−^ mice (5‐month‐old) showed there was increased activation of PDGFR‐α^+^ cells and CD68^+^ cells (macrophages) and decreased activation of Pax7^+^ cells (Figure 1a–d) compared to age‐matched WT controls. qPCR analysis of PDGFR‐α in muscle tissues further verified the increased expression of PDGFR‐α in *Z24* ^−/−^ muscle (Figure 1e). Consistently, the skeletal muscle of *Z24* ^−/−^ mice had increased fibrosis and cellular senescence and impaired muscle regeneration capacity (Figure S1). In both WT and *Z24* ^−/−^ muscle, PDGFR‐α^+^ cells localize adjacent to Pax7^+^ muscle stem cells at the muscle stem cell niche among myofibers (Figure 1f), suggesting that PDGFR‐α^+^ cells could impact Pax7^+^ cells through the release of soluble factors or through direct cell–cell interaction. Therefore, we examined the effect of mesenchymal stromal/stem cells (MSCs) isolated from the skeletal muscles of WT and *Z24* ^−^ *^/^* ^−^ mice on WT muscle progenitor cells (MPCs).

Immunostaining of MSCs demonstrated that both *Z24* ^−/−^ and WT MSCs were predominantly PDGFR‐α positive (Figure 1g). qPCR analysis further demonstrated that *Z24* ^−^ *^/^* ^−^ MSCs specifically expressed higher level of senescence and SASP markers (i.e., p16^INK4a^, CXCL1, MCP1, IL‐1α, IL‐1β, IL‐6, and TNFR1) and the pro‐fibrotic factor TGF‐β1 (Figure 1h), whereas the expression of anti‐inflammatory factors (i.e., IL‐10 and Klotho) was down‐regulated. In addition, conditioned medium (CM) from *Z24* ^−^ *^/^* ^−^ MSCs was able to confer repression of the myogenic potential of WT MPCs (Figure 1i) and increase the percentage of cells with damaged DNA (γ‐H2AX^+^) (Figure 1i). The expression of pro‐inflammatory factors (i.e., IL‐6, and TNF‐α) and pro‐fibrogenic factors (i.e., TGF‐β1, Collagen I, PDGFR‐α, PDGFR‐β) was up‐regulated in WT MPCs, whereas the expression of anti‐inflammation factors (i.e., IL‐10, and Klotho) was down‐regulated by cultivating the WT MPCs with CM from *Z24* ^−^ *^/^* ^−^ MSCs (Figure 1j).

### 2.2 ***MSCs from muscle of Z24*** ^−/−^ ***mice exhibit increased nuclear abnormalities and cytoskeletal stiffness***

**Figure 2:**
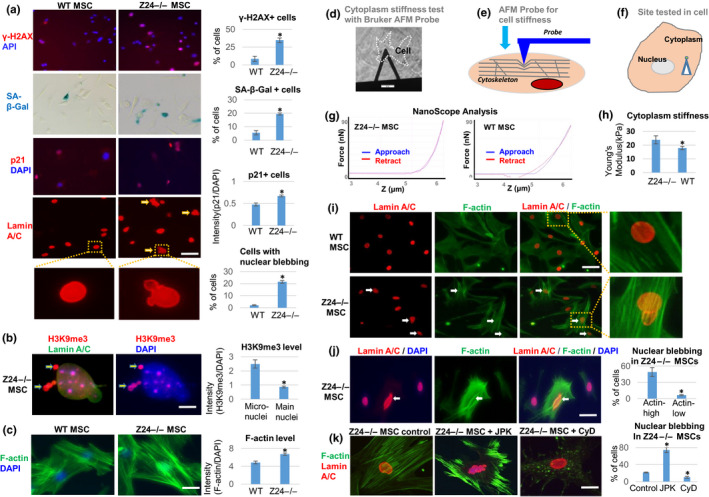
*Z24* ^−/−^ MSCs display increased senescent phenotypes, and enhanced F‐actin polymerization and cytoskeletal stiffness is directly associated with increased and nuclear blebbing. MSCs isolated from the skeletal muscle of WT and *Z24* ^−/−^ mice were compared. (a) Immunostaining analysis of γ‐H2AX, p21^Cip1^, and lamin A/C was performed, as well as SA‐β‐Gal staining for senescence. Quantitation of γ‐H2AX^+^ cells, SA‐β‐Gal^+^ cells, p21^+^ cells, and cells with nuclear blebbing is shown. Scale bar = 30µm. (b) Immunostaining analysis and quantification of H3K9me3. The increased level of H3K9me3 (red) in the micronuclei in contrast to nucleus indicates the loss of heterochromatin from nucleus to micronuclei (arrows). Scale bar = 2.5 µm. (c) Staining of F‐actin with Alexa Fluor 488 Phalloidin and quantification of F‐actin polymerization. Scale bar = 20 µm. (d–g). Testing of cytoplasm stiffness using a Bruker AFM probe. H. The cytoplasm stiffness (kPa) calculated by NanoScope analysis. (i) Immunostaining analysis of lamin A/C and F‐actin in WT and *Z24* ^−/−^ MSCs, showing higher level of F‐actin and nuclear blebbing in same *Z24* ^−/−^ cell (arrow). Scale bar = 50 µm. (j) Immunostaining analysis of lamin A/C and F‐actin in *Z24* ^−/−^ MSCs. Quantitation of nuclear blebbing is shown. The number of cells with nuclear blebbing was compared between cells with top 30% of F‐actin intensity (Actin‐high) and cells with bottom 30% of F‐actin intensity (Actin‐low). Scale bar = 30 µm. (k) Immunostaining analysis of lamin A/C and F‐actin to observe the effect of treatment of *Z24* ^−/−^ MSCs with F‐actin stabilizing JPK (200 nM) or F‐actin depolymerizing CyD (100 ng/ml) for 48 hr. Quantitation of nuclear blebbing is shown. Scale bar = 15 µm. Arrows: nuclear blebbing. *N *≥ 6. “*” at bar charts indicates *p* < .05

*Z24* ^−^ *^/^* ^−^ MSCs developed increased DNA damage (γ‐H2AX^+^) and cellular senescence (SA‐β‐Gal^+^) (Figure 2a), which are consistent to the *Z24* ^−^ *^/^* ^−^ mice progeria phenotypes. p21^Cip1^, a protein associated with cellular senescence (Calio et al., 2015), was increased in *Z24* ^−^ *^/^* ^−^ MSCs (Figure 2a). Abnormal nuclear morphology and protrusions termed “blebs” are diagnostic markers for aging and progeria and are key features of the HGPS cell nucleus (Capell et al., 2005). Immunostaining of Lamin A/C proteins was performed to examine nuclear morphology, revealing increased nuclear blebbing and nuclear irregularity in *Z24* ^−^ *^/^* ^−^ MSCs compared to WT MSCs (Figure 2a; Figure S2).

Recently, cytoplasmic chromatin fragments (CCF) present in the micronuclei derived from the main nucleus were shown to trigger cGAS‐Sting innate immune signaling and cellular senescence (Dou et al., 2017). Progerin and telomere dysfunction both can trigger cellular senescence by inducing chromatin‐carrying micronuclei (Cao et al., 2011). We observed the presence of cytoplasmic chromatin fragments [H3K9me3‐positive heterochromatin (Shumaker et al., 2006)] in the micronuclei of *Z24* ^−/−^ MSCs (Figure 2b). We also observed intensive condensation of telomeres and translocation of telomeres into micronuclei (Figure S3), which confirms the cytoplasmic localization of chromatin fragments in *Z24* ^−/−^ MSCs. Importantly, using phalloidin staining of F‐actin, we also observed increased F‐actin polymerization in *Z24* ^−/−^ MSCs (Figure 2c; Figure S4a,b), suggesting a potentially increased cytoskeleton stiffness in these cells. In addition, the level of total actin was not significantly different between WT MSCs and *Z24* ^−/−^ MSCs (Figure S4c,d), suggesting that the dynamic F‐actin formation from G‐actin in *Z24* ^−/−^ MSCs is responsible for the increased F‐actin polymerization, rather than the increased production of new actin protein.

To further confirm this observation, cell stiffness was tested by the atomic force microscopy (AFM) system (Figure 2d,h). The Young modulus force of *Z24* ^−/−^ MSCs was significantly higher than WT MSCs (Figure 2h), especially in cells with nuclear blebbing, demonstrating a positive correlation of cell stiffness and nuclear blebbing.

### 2.3 ***F‐actin polymerization and nuclear abnormalities are closely coupled in Z24*** ^−/−^ ***MSCs***

We further validated that *Z24* ^−/−^ MSCs exhibited both increased F‐actin polymerization and higher rate of nuclear blebbing by co‐staining F‐actin and lamin A/C (Figure 2i). Within *Z24* ^−/−^ MSCs, the increased F‐actin polymerization in individual cells was directly coupled with a higher rate of nuclear blebbing (Figure 2j). In order to verify the direct effect of F‐actin polymerization on nuclear blebbing, cytoskeletal stiffness was modified by stabilizing or destabilizing F‐actin in *Z24* ^−/−^ MSCs. *Z24* ^−/−^ MSCs were treated with jasplakinolide (JPK) to induce F‐actin stabilization or with cytochalasin D (CyD) to induce F‐actin depolymerization. An increase in nuclear blebbing in *Z24* ^−/−^ MSCs was observed upon JPK treatment, whereas CyD treatment decreased nuclear blebbing (Figure 2k). This result further suggests that the increased cytoskeletal stiffness developed by sustained polymerization of F‐actin can contribute to nuclear blebbing.

In order to further determine whether F‐actin polymerization can induce a senescent phenotype in progeria cells and whether releasing the mechanical tension of F‐actin cytoskeleton can rescue the senescent phenotypes, we treated *Z24* ^−/−^ MSCs with JPK or CyD and examined the expression of genes associated with cellular senescence. JPK treatment increased expression of p16^INK4a^, p21^Cip1^, IL1‐β, and TNFR1. In contrast, expression of p16^INK4a^, p21^Cip1^, IL1‐β, and TNFR1 was down‐regulated after CyD treatment of *Z24* ^−/−^ MSCs, whereas expression of IL‐10 was up‐regulated (Figure S5).

### 2.4 ***RhoA is activated in Z24*** ^−/−^ ***MSCs and is coupled with increased nuclear blebbing and cellular senescence***

**Figure 3:**
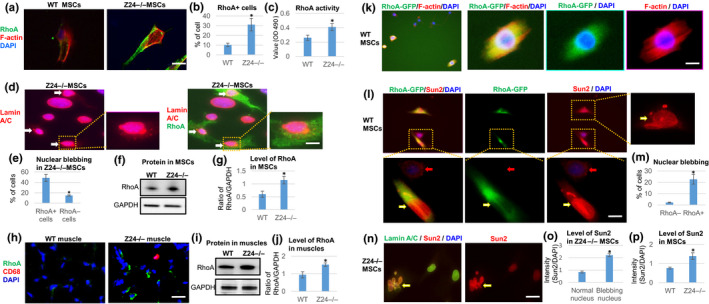
Increased RhoA activation in *Z24* ^−/−^ MSCs, and effect of RhoA over‐expression on Sun2 and nuclear blebbing in WT MSCs. (a) Immunostaining analysis of RhoA and F‐actin in WT and *Z24* ^−/−^ MSCs. Scale bar = 30 µm. (b) Quantification of RhoA^+^ cells is shown. (c) Quantification of RhoA activity is shown. (d) Immunostaining analysis of RhoA and lamin A/C in *Z24* ^−/−^ MSCs. Arrows: cells with higher RhoA expression and nuclear blebbing. Scale bar = 5 µm. (e) Quantification of nuclear blebbing in RhoA^+^ and RhoA‐ *Z24* ^−/−^ MSCs is shown. (f, g) Western blot analysis and quantification of RhoA in WT and *Z24* ^−/−^ MSCs, with GAPDH as loading control. (h) Immunostaining analysis of RhoA^+^ cells and CD68^+^ inflammatory cells in skeletal muscle of *Z24* ^−/−^ mice. (i, j) Western blot analysis and quantification of RhoA in muscle tissues from WT and *Z24* ^−/−^ mice, with GAPDH as loading control. (k) WT MSCs were transfected with a plasmid carrying constitutively active RhoA‐GFP and stained for F‐actin. Scale bar = 5 µm. (l) Immunostaining analysis of Sun2 to check Sun2 and nuclear blebbing in RhoA‐GFP transfected WT MSCs. Yellow arrows: cells with RhoA‐GFP; red arrows: cells without RhoA‐GFP. Scale bar = 5 µm. (m) Quantification nuclear blebbing (RhoA‐GFP‐ V.S. RhoA‐GFP^+^ cells) is shown. (n) Immunostaining analysis of Sun2 and lamin A/C in *Z24* ^−/−^ MSCs. Scale bar = 10µm. (o) Quantification of Sun2 in *Z24* ^−/−^ MSCs without or without nuclear blebbing is shown. (p) Quantification of Sun2 in WT and *Z24* ^−/−^ MSCs is shown. *N *≥ 6. “*” at bar charts indicates *p* < .05

F‐actin polymerization requires the activation of RhoA, and RhoA can be activated by DNA damage (Aghajanian, Wittchen, Campbell, & Burridge, 2009). Since there is both increased F‐actin polymerization and DNA damage in *Z24* ^−/−^ MSCs, RhoA signaling was examined in *Z24* ^−/−^ MSCs. Immunostaining analysis showed that the ratio of RhoA^+^ cells was higher in *Z24* ^−/−^ MSCs than WT MSCs and increased RhoA in the *Z24* ^−/−^ MSCs is coupled with increased F‐actin polymerization (Figure 3a,b). Consistently, there was higher RhoA activity in *Z24* ^−/−^ MSCs in contrast to WT MSCs (Figure 3c). There was also an increased ratio of nuclear blebbing in RhoA^+^ cells (Figure 3d,e). Western blot analysis also demonstrated a higher level of RhoA in *Z24* ^−/−^ MSCs (Figure 3f,g). Furthermore, there was increased activation of RhoA^+^ cells in the skeletal muscle of *Z24* ^−/−^ mice as analyzed by both immunohistochemistry and Western blot analysis (Figure 3h‐i).

### 2.5 Constitutive activation of RhoA signaling promotes nuclear abnormalities and cellular senescence

In order to verify further the role of RhoA activation in modulating nuclear abnormalities and cellular senescence, the effect of RhoA activation in WT MSCs was examined. Treatment of WT MSCs with the Rho activator II (Cytoskeleton Inc.), which increases the level of GTP‐bound RhoA (Schmidt et al., 1997), increased F‐actin polymerization and nuclear blebbing (Figure S6). Similarly, WT MSCs transfected with a plasmid carrying constitutively active RhoA‐GFP developed increased F‐actin polymerization in GFP‐positive cells (Figure 3k). The level of nuclear blebbing also was found to be higher in RhoA‐GFP^+^ cells than RhoA‐GFP‐ cells (Figure 3l,m). Sun proteins (i.e., Sun1 and Sun2) at the nuclear envelope transduce mechanical stress from the ECM and cytoskeleton into the nucleus (Lei et al., 2009). LINC complexes that contain Sun2, but not Sun1, were found to promote focal adhesion assembly by activating RhoA (Thakar et al., 2017). Thus, we examined whether Sun2 may mediate the effect of RhoA activation in promoting nuclear blebbing in *Z24* ^−/−^ MSCs. We observed that the expression of constitutively active RhoA‐GFP in WT MSCs elevated Sun2 protein level (Figure 3l). Meanwhile, we observed that Sun2 was not expressed uniformly in *Z24* ^−/−^ MSCs and appeared to be more prominently expressed in cells with nuclear blebbing (Figure 3n,o; Figure S7) and increased F‐actin polymerization (Figure S7a). Consistently, the level of Sun2 was generally higher in *Z24* ^−/−^ MSCs than WT MSCs (Figure 3p).

In WT MSCs, the level of Sun2 also was increased by JPK treatment (Figure S8a), further suggesting that Sun2 is responsive to the changes in F‐actin cytoskeleton stiffness and nuclear stiffness. Western blot analysis of Sun1 and Sun2 in WT MSCs, with or without treatment with Rho activator II or JPK, and *Z24* ^−/−^ MSCs showed that the level of Sun2, but not Sun1, was elevated by RhoA activation and F‐actin stabilization (Figure S8b). Similarly, we observed increased number of cells with higher Sun2 expression in the skeletal muscle tissues of *Z24* ^−/−^ mice (Figure S9).

### 2.6 ***Inhibition of RhoA signaling in Z24*** ^−/−^ ***MSCs reduced F‐actin polymerization, nuclear blebbing, DNA damage, and cellular senescence***

**Figure 4:**
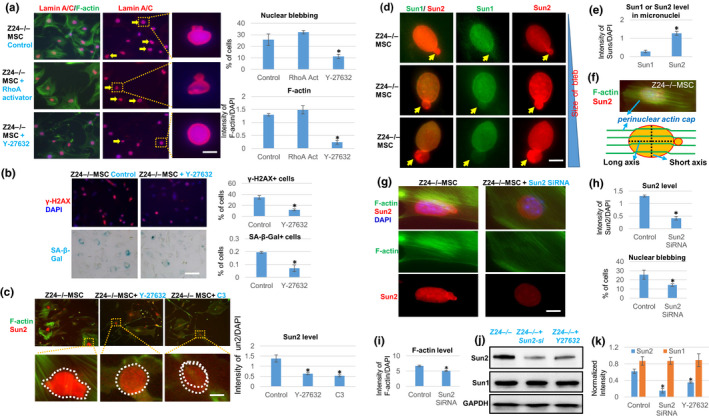
Effect of inhibition of RhoA/ROCK signaling or Sun2 expression in *Z24* ^−/−^ MSCs. (a) Immunostaining analysis of lamin A/C and F‐actin in *Z24* ^−/−^ MSCs treated with Rho activator II or RhoA/ROCK inhibitor Y‐27632. Quantification of nuclear blebbing and F‐actin is shown. Scale bar = 5 µm. (b) Immunostaining analysis and quantification of γ‐H2AX and SA‐β‐Gal staining in *Z24* ^−/−^ MSCs treated with Y‐27632. Quantification of γ‐H2AX^+^ or SA‐β‐Gal^+^ cells is shown. Scale bar = 100 µm. (c) Immunostaining analysis of Sun2 and F‐actin in *Z24* ^−/−^ MSCs treated with Y‐27632 or C3 transferase (C3). Quantification of Sun2 with or without RhoA inhibition is shown. Scale bar = 3 µm. (d) Immunostaining analysis of Sun1 and Sun2 in nuclear and micronuclei of *Z24* ^−/−^ MSCs. Scale bar = 3 µm. (e) Quantitation of Sun1 and Sun2 protein level in micronuclei of *Z24* ^−/−^ MSCs is shown. (f) Demonstration of perinuclear actin cap stress fiber. (g) Immunostaining analysis of Sun2 and F‐actin in *Z24* ^−/−^ MSCs with or without Sun2 SiRNA treatment. Scale bar = 3 µm. (h) Quantitation of Sun2 and nuclear blebbing is shown. (i) Quantification of F‐actin level is shown. (j) Western blot analysis of Sun1 and Sun2 in *Z24* ^−/−^ MSCs and *Z24* ^−/−^ MSCs treated with Y‐27632 or Sun2 SiRNA. (k) Quantitation of Sun1 and Sun2 in western blot result is shown. *N *≥ 6. “*” at bar charts indicates *p* < .05

Based on the results above, we hypothesized that inhibition of RhoA signaling could suppress senescent phenotypes in progeria cells by reducing polymerized F‐actin and relaxing the cytoskeleton stiffness. *Z24* ^−/−^ MSCs treated with RhoA/ROCK inhibitor Y‐27632 showed a decrease in F‐actin polymerization and nuclear blebbing in contrast to *Z24* ^−/−^ MSCs and *Z24* ^−/−^ MSCs treated with Rho activator II (Figure 4a). Also, Y‐27632 treatment reduced the level of the DNA damage marker γ‐H2AX^+^ and the number of senescent cells (SA‐β‐Gal+ or p21^Cip1^+) (Figure 4b; Figure S10). Y‐27632 treatment of *Z24* ^−/−^ MSCs also reduced the translocation of telomeres into micronuclei and the condensation of telomere (Figure S10b). The specificity of Y‐27632 in inhibiting RhoA/ROCK signaling in *Z24* ^−/−^ MSCs was further verified by measuring the activity of RhoA and ROCK in *Z24* ^−/−^ MSCs with or without Y‐27632 treatment (Figure S10c). Importantly, Y‐27632 treatment of *Z24* ^−/−^ MSCs also reduced the level of Sun2 protein, which is correlated with reduced nuclear blebbing and F‐actin polymerization (Figure 4c; Figure S11a). C3 transferase, a specific inhibitor targeting RhoA (Gutekunst, Tung, McDougal, & Gross, 2016), also reduced F‐actin polymerization and Sun2 expression in *Z24* ^−/−^ MSCs (Figure 4c). Moreover, the testing of cell stiffness with AFM system showed that Y‐27632 treatment reduced the nuclear and cytoskeletal stiffness of *Z24* ^−/−^ MSCs (Figure S14a–c). These results suggest that increased sun2 expression in *Z24* ^−/−^ MSCs plays a crucial role in regulating cytoskeleton stiffness.

### 2.7 ***Effect of repressing Sun2 expression with Sun2 SiRNA in Z24*** ^−/−^ ***MSCs***

We observed higher Sun2 expression in micronuclei of *Z24* ^−/−^ MSCs than Sun1 (Figure 4d,e; Figure S12). In order to examine the role of Sun2 further, *Z24* ^−/−^ MSCs were transfected with Sun2 siRNA to repress Sun2 expression. Reduced Sun2 expression disrupted the aligned structure of the perinuclear actin cap stress fiber (Figure 4f,g) and reduce the F‐actin level at the perinuclear actin cap (Khatau, Kim, Hale, Bloom, & Wirtz, 2010) (Figure S13). Also, reduced Sun2 expression decreased nuclear blebbing in *Z24* ^−/−^ MSCs (Figure 4g,h) and the level of total F‐actin (Figure 4i). Moreover, the testing of cell stiffness with AFM system showed that the Young modulus force of *Z24* ^−/−^ MSCs treated with Sun2 SiRNA or Y‐27632 was reduced, at both the nuclear/cytoskeletal and cytoskeletal locations (Figure S14a–c). In addition, treatment of *Z24* ^−/−^ MSCs treated with a Sun2 SiRNA reduced RhoA activity (Figure S14d) and down‐regulated expression of senescence‐associated genes (Figure S14e). In addition, treatment of *Z24* ^−/−^ MSCs with either Sun2 SiRNA or Y‐27632 reduced the level of Sun2 protein, but not Sun1 protein (Figure 4j,k).

### 2.8 ***Inhibition of RhoA/ROCK repressed cGAS/Sting signaling in Z24*** ^−/−^ ***MSCs***

**Figure 5:**
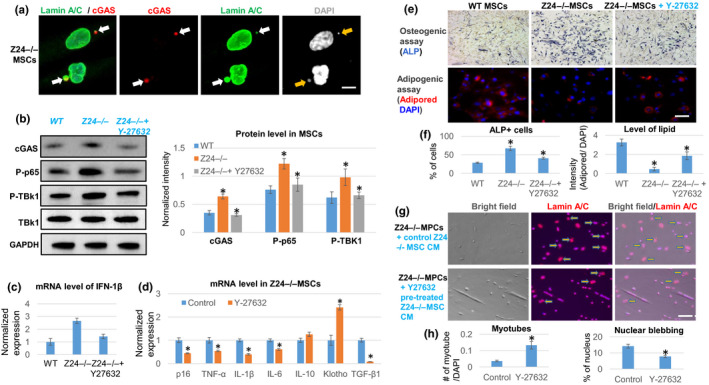
RhoA inhibition in *Z24* ^−/−^ MSCs represses micronuclei/cytoplasmic DNA‐induced innate immune response, reduces SASP expression, and rescues senescent phenotypes. (a) Immunostaining analysis of lamin A/C and cGAS showed that there is positive cGAS deposition at the micronuclei formed in *Z24* ^−/−^ MSCs (arrows). Scale bar = 3 µm. (b) Western blot analysis and quantification of proteins related to the cGAS‐Sting signaling (cGAS, phosphor‐p65, phosphor‐TBK1) in WT MSCs,* Z24* ^−/−^ MSCs, and *Z24* ^−/−^ MSCs treated with Y‐27632. (c) qPCR analysis of interferon‐1β (IFN‐1β) expression. (d) qPCR analysis of the expression of SASP and senescent‐associated genes in *Z24* ^−/−^ MSCs with or without Y‐27632 treatment. (e) Osteogenesis assay and adipogenesis assay of *Z24* ^−/−^ MSCs with or without Y‐27632 treatment. Osteogenic potential was examined with ALP staining of osteogenic cells, and adipogenic potential was examined with AdipoRed staining of lipid in adipogenic cells. Scale bar = 30 µm. (f) Quantification of ALP or AdipoRed is shown. (g) Immunostaining analysis of lamin A/C in *Z24* ^−/−^ MPCs treated with conditioned medium from *Z24* ^−/−^ MSCs with or without Y‐27632 pretreatment. Arrows indicate cells with nuclear blebbing. Scale bar = 50 µm. (h) Quantification of myotube number and nuclear blebbing is shown. *N *≥ 6. “*” at bar charts indicates *p* < .05

Micronuclei formation is a danger signal to the cells, leading to activation of the cGAS‐Sting signaling pathway, a component of the innate immune system, to promote cellular senescence (Dou et al., 2017). The micronuclei formed in *Z24* ^−/−^ cells are positive for cGAS protein by immunostaining assay (Figure 5a). In addition, treatment of the *Z24* ^−/−^ cells with Y‐27632 was able to reduce the protein level of cGAS, phospho‐p65/RelA, and phopho‐TBK1 in *Z24* ^−/−^ cells (Figure 5b). Also, Y‐27632 treatment down‐regulated the expression of the type 1 interferon‐β (IFN‐1β), a marker of activation of cGAS‐Sting innate immune signaling (Figure 5c).

### 2.9 ***Inhibition of RhoA/ROCK signaling in Z24*** ^−/−^ ***MSCs repressed SASP expression, modified the differentiation potential, and rescued the deleterious effect of Z24*** ^−/−^ ***MSCs on MPCs***

Hutchinson–Gilford progeria syndrome MSCs have decreased adipogenic potential and increased osteogenic potential (Meshorer & Gruenbaum, 2008). qPCR analysis revealed that the expression of SASP factors (i.e., IL‐1β, IL‐6, and TNF‐α), p16^INK4a^, and TGF‐β1 was down‐regulated upon Y‐27632 treatment of *Z24* ^−/−^ MSCs, whereas the expression of the anti‐inflammation factors IL‐10 and Klotho was up‐regulated (Figure 5d). Also, Y‐27632 treatment of *Z24* ^−/−^ MSCs was able to restore the adipo‐osteogenic balance by promoting adipogenesis and repressing osteogenesis (Figure 5e,f). In addition, when the *Z24* ^−/−^ MPCs were treated with conditioned medium from *Z24* ^−/−^ MSC pretreated with Y‐27632, the myogenic potential of *Z24* ^−/−^ MPCs was increased and the number of cells with nuclear blebbing was reduced, compared to *Z24* ^−/−^ MPCs treated with control conditioned medium from *Z24* ^−/−^ MSCs without Y‐27632 pretreatment (Figure 5g,h).

### 2.10 ***Inhibition of RhoA/ROCK promotes epigenetic changes in chromatin of Z24*** ^−/−^ ***MSCs***

HGPS cells have profound alterations in epigenetics and histone modification with reduced heterochromatin markers H3K9me3 and H3K27me3 (Arancio, Pizzolanti, Genovese, Pitrone, & Giordano, 2014). We examined whether the inhibition of RhoA/ROCK activity or Sun2 expression affected the epigenetic state of chromatin in *Z24* ^−/−^ MSCs. H3K9me3 and H3K27me3 were higher in cells without obvious nuclear blebbing in contrast to cells with nuclear blebbing (Figure S15a). Also, both Y‐27632 and Sun2 siRNA were effective in increasing the level of H3K9me3 or H3K27me3 in *Z24* ^−/−^ MSCs (Figure S15b–d).

### 2.11 ***Systemic inhibition of RhoA in Z24*** ^−/−^ ***mice extended the healthspan and rescued the defective phenotypes in skeletal muscle***

**Figure 6:**
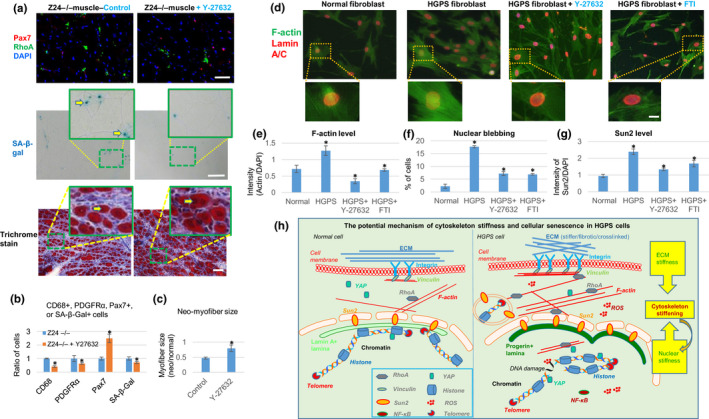
Effect of RhoA inhibition in *Z24* ^−/−^ mice in vivo, and effect of RhoA inhibition in HGPS fibroblasts. (a) Systemic inhibition of RhoA/ROCK signaling in *Z24* ^−/−^ mice was performed by i.p. injection of Y‐27632. Immunostaining analysis of Pax7 and RhoA and SA‐β‐Gal staining of senescent cells was performed with muscle tissues from WT and *Z24* ^−/−^ mice. Trichrome staining was performed with regenerating muscle tissues (7 days after cardiotoxin‐induced muscle injury). Scale bars = 100 µm. (b) Quantitation of the changes in CD68^+^, PDGFR‐α^+^, Pax7^+^, and SA‐β‐Gal^+^ cells in muscles of *Z24* ^−/−^ mice with or without Y‐27632 injection are shown. (c) Quantification of the size of regenerating neo‐myofibers (positive with centrally located nuclei). (d) Immunostaining analysis of lamin A/C and F‐actin in normal human fibroblasts, HGPS fibroblasts, and HGPS fibroblasts treated with Y‐27632 or farnesyltransferase inhibitor (FTI). Scale bar = 5µm. (e) Quantitation of F‐actin is shown. (f) Quantitation of nuclear blebbing is shown. (g) Quantitation of Sun2 is shown. (h) The proposed model for the potential mechanism of RhoA/Sun2‐mediated increase of cytoskeletal stiffness in progeria cells. The ECM, cytoskeleton and nucleoskeleton are physically connected. Our results suggest that both increased ECM stiffness and nuclear stiffness of progeria cells contribute to increased RhoA activation and cytoskeleton stiffness. Also, elevated ROS production, inflammatory signaling (NF‐κB), and DNA damages in progeria cells can promote RhoA activation, resulting in further increased cytoskeleton stiffness. Increased RhoA and Sun2 expression, nuclear blebbing, and cytoplasmic DNA fragments in micronuclei, can all mediate accelerated cellular senescence. *N *≥ 6. “*” at bar charts indicates *p* < .05

To examine the effect of RhoA inhibition in vivo, 10‐week‐old *Z24* ^−/−^ mice were treated with Y‐27632 (10 mg/kg) by i.p. injection 3 times a week for 12 weeks. Following this treatment regimen, the number of myogenic Pax7^+^ cells was increased in the skeletal muscles of *Z24* ^−/−^ mice (Figure 6a,b), whereas the number of SA‐β‐Gal^+^ cells, pro‐inflammatory CD68^+^ cells, and PDGFR‐α^+^ cells was reduced (Figure 6b). Also, RhoA inhibition improved the muscle regeneration potential of *Z24* ^−/−^ mice following cardiotoxin‐induced injury (7 days after injury) (Figure 6a,c). This suggests that RhoA inhibition improves the function of muscle stem cells and muscle regeneration both by increasing the number of muscle stem cells and repressing the deleterious impact of senescent cells on muscle stem cells. Based on these results, we propose a potential cellular and molecular mechanism of muscle stem cell dysfunction in *Z24* ^−/−^ mice, involving the over‐activation of RhoA signaling, and pro‐inflammatory, and pro‐fibrogenic factors in MSCs and inflammatory cells (Figure S16).

### 2.12 Human HGPS fibroblasts have increased F‐actin polymerization, which was reduced by RhoA/ROCK inhibitor and farnesyltransferase inhibitor (FTI)

To extend the results from the *Z24* ^−/−^ mouse model of HGPS, fibroblasts from HGPS patients also were examined. Similar to murine *Z24* ^−/−^ cells, there was increased F‐actin polymerization and nuclear blebbing (Figure 6d) in human HGPS fibroblasts compared to normal fibroblasts. Treatment of HGPS fibroblasts with Y‐27632 reduced F‐actin polymerization, nuclear blebbing, and Sun2 expression (Figure 6d–g). Furthermore, both H3K9me3 and H3K27me3 were generally less prevalent in HGPS fibroblasts with nuclear blebbing (Figure 6h), whereas treatment of HGPS cells with Y‐27632 resulted in increased levels of H3K9me3 and H3K27me3 (Figure S17). In addition, AFM probe testing of cell stiffness showed that HGPS fibroblasts seemed to have a higher cytoskeletal stiffness than normal fibroblasts (Figure S18).

The farnesyltransferase inhibitor (FTI) lonafarnib currently is the only approved medication for HGPS patients, effective in reversing the dramatic nuclear structure abnormalities and to alleviate some disease symptoms. FTIs act by inhibiting the farnesylation of prelamin A (Mehta, Eskiw, Arican, Kill, & Bridger, 2011). Intriguingly, the activity of RhoA protein also is regulated by farnesylation. This common farnesylation‐dependent regulatory mechanism of both prelamin A and RhoA proteins suggests that RhoA might serve as a promising target for therapeutic treatment of HGPS. We observed that treatment of HGPS fibroblasts with lonafarnib efficiently reduced F‐actin polymerization, nuclear blebbing, and Sun2 expression (Figure 6d–g).

## 3 DISCUSSION

The *LMNA* mutation in HGPS disease causes nuclear abnormalities and cellular fragility in response to mechanical stress; however, correlations between genotypes and mechanical phenotypes in the cells remain unclear. Also, the role of cytoskeletal stiffness and its potential association with nuclear abnormality in progeria cells also remains to be investigated. Progerin production occurs not only in progeria cells, but also in naturally aged human cells (Booth et al., 2015; Cao et al., 2011; Pacheco et al., 2014; Phillip et al., 2015). The role of cytoskeletal stiffness in mediating the deleterious effect of progerin in promoting cellular senescence and aging is poorly understood. Therefore, we examined the mechanism of cytoskeletal stiffness‐mediated nuclear variations in HGPS cells and in a HGPS mouse model. Here, we demonstrate the crucial role of cytoskeletal stiffness in mediating nuclear abnormalities in cells from HGPS patients and a progeria disease mouse model as well as the mechanical roles of RhoA and Sun2 in modulating cytoskeletal stiffness, nuclear blebbing, and senescence.

Our results are consistent with previous reports of increased cytoskeletal stiffness in certain types of senescent cells, which also suggests that cytoskeletal stiffness is coupled with nucleoskeletal stiffness (Nishio, Inoue, Qiao, Kondo, & Mimura, 2001; Schulze et al., 2010). Our results further indicate that activation of the RhoA signaling and Sun2 protein is crucial for mediating the accelerated cellular senescence of MSCs and aging‐associated pathologies in the muscle of HGPS patients. Interestingly, a recent chemical screening study identified ROCK as a target for recovering mitochondrial function in HGPS cells. Here, Y‐27632 treatment of HGPS cells induced the recovery of mitochondrial function and reduction of abnormal nuclear morphology (Kang et al., 2017), which is consistent with our results. However, our study further revealed the biomechanical mechanism of RhoA/ROCK activation in mediating cellular senescence, including the direct connection of RhoA/ROCK activation with cell stiffness and nuclear blebbing, the interaction of RhoA/ROCK and Sun2 in response to cytoskeleton stiffness and nuclear blebbing, and the correlation among RhoA/ROCK activation, Sun2 expression, micronuclei formation, and the innate (cGAS/Sting) immune response.

There have been previous reports describing the normal cytoskeletal stiffness in HGPS cells (Verstraeten et al., 2008) and reduced cytoskeletal stiffness in LMNA mutant (*LmnaL530P*/*L530P*) mouse cells (Hale et al., 2008). These different results in cytoskeletal stiffness compared to our studies may be partially attributed to the different cell lineages, cell culture conditions, or stiffness testing methods. *LmnaL530P*/*L530P* cells do not produce progerin, and there is a decreased level of lamin A in nuclear lamina (Hale et al., 2008). The weaker/softer nuclear lamina in *LmnaL530P*/*L530P* cells could be responsible for the reduced cytoskeleton stiffness observed. However, LMNA mutation in HGPS cells leads to progerin accumulation and trapping of lamin A/C in the nuclear lamina, forming orientationally ordered microdomains and stiffer lamina network (Dahl et al., 2006).

Our results reveal the mechanical mechanism of how RhoA/ROCK activation promotes cellular senescence by modulating F‐actin polymerization in progeria cells. The increased F‐actin polymerization and cytoskeletal stiffness in senescent cells could be caused by sustained RhoA activation, which can impair proper actin dynamics and normal cellular function, and further enhance DNA damage, ROS production, nuclear blebbing, cytoplasmic DNA/cGAS‐Sting‐mediated innate immune responses, chromatin dysfunction, and other progeria phenotypes. Thus, inhibition of RhoA activation potentially rescues progeria phenotypes by relieving cytoskeletal stiffness and consequently nucleoskeletal stiffness. Our results also suggest that the accelerated DNA damage and cellular senescence in progeria cells can be delayed by simply reducing cytoskeletal stiffness. In addition, systemic inhibition of RhoA/ROCK signaling in progeria mice was able to rescue the progeria phenotypes in skeletal muscle.

We propose that RhoA activation is an indirect outcome of the *LMNA* mutation in progeria cells through two possible mechanisms. The first mechanism involves the elevated cytoskeletal mechanical stress induced by mutation of *LMNA*. This leads to progerin accumulation in nuclear lamina, which increases the stiffness of lamina network (Dahl et al., 2006). This mechanical stress from lamina network can be transmitted to cytoskeleton via the LINC complex, and mechanical sensing factors in the cytosol can mobilize RhoA activation to promote F‐actin polymerization. The second possibly mechanism involves the increased Sun2 expression induced by *LMNA* mutation. Recent studies indicates that Sun2 is mechanical responsive (Hoffman et al., 2020), and the expression of Sun2 in LINC complex can be modulated by varied mechanical features of lamina network. Sun2 was found to promote RhoA activation (Thakar et al., 2017), and thus, the *LMNA* mutation may cause RhoA activation by increasing Sun2 expression in HGPS cells.

Previous observations have demonstrated the role of Sun1 in responding to progerin accumulation in nuclear lamina (Haque et al., 2010; Lei et al., 2009). It was shown that the reduction of Sun1 accumulation in LMNA mutant cells (i.e., *Lmna^−/−^* and *Lmna*Δ9 fibroblasts) corrected nuclear defects and cellular senescence (Chen et al., 2012). We believe that this beneficial effect of Sun1 reduction in progeria cells may be partially due to the regulation of mechanical features of nucleus or cytoskeleton since nuclear lamina network is connected to cytoskeleton by both Sun1 and Sun2 in the LINC complexes. However, we did not observe the accumulation of Sun1 in our cells, potentially because of the different cell culture condition and genotypes of cell lineages, especially the potential difference in progerin generation in cells. Recent studies have suggested a potential role for Sun2 in mechano‐sensing and involvement in mechanical stimulation‐induced changes in actin cytoskeleton (Hoffman et al., 2020). Our results have confirmed further the mechanical role of Sun2 in mediating increased nuclear stiffness, nuclear abnormalities, and cellular senescence, as well as a potential mechanical feedback loop of Sun2 with RhoA.

In aged tissues, the extracellular matrix (ECM) usually becomes stiffer because of increased collagen deposition, varied composition of collagen subtypes, and the crosslinking structure of collagen fiber. Cells adapt to this increased ECM stiffness by modifying the expression of mechano‐responsive genes. Mechanical stimuli from the ECM can be transmitted to the nucleus via the cytoskeleton and LINC complex, and directly contribute to the triggering of mechano‐responsive genes to adapt to the mechanical environment (Isermann & Lammerding, 2013; Phillip et al., 2015). Both lamin and Sun proteins are required for this structural connection between the nucleus and cytoskeleton (Isermann & Lammerding, 2013; Phillip et al., 2015) and for transducing mechanical stresses from ECM and cytoskeleton into nucleus, which is essential for a broad range of cellular functions. Furthermore, the expression of both lamin A and Sun2 was found to be mechano‐responsive and scales with ECM/substrate stiffness or elasticity. A stiff substrate is associated with increased lamin A and Sun2 expression and strengthened nuclear envelope, whereas a soft substrate for cells is associated with reduced lamin A and Sun2 expression. In aged and progeroid cells, progerin accumulation in the nuclear lamina causes increased nuclear stiffness (Booth et al., 2015; Cao et al., 2011; Dahl et al., 2006; Phillip et al., 2015; Verstraeten et al., 2008; Young et al., 2005). Our current results with *Z24* ^−/−^ MSCs and HGPS fibroblasts demonstrate the increased accumulation of Sun2 protein in the nuclear envelope, especially in cells with abnormal nuclear architecture (nuclear blebbing). The increased activation of Sun2 in these in vitro cultured progeria cells is not caused by stiffer substrate/ECM, but rather is a result of an intrinsic change in the mechanical properties of the cell nucleus. We propose that Sun2 accumulation in progeria cells is a result of progerin accumulation because there is direct physical interaction between the Sun2 and lamin A proteins. Mechanical stimuli resulting from increased nuclear stiffness could be transmitted to the cytoskeleton via LINC, leading to increased F‐actin polymerization. The Sun2 protein also can activate RhoA signaling directly (Thakar et al., 2017), which may further enhance F‐actin polymerization and cytoskeletal stiffness. Therefore, increased cytoskeletal stiffness in progeria cells could actually be the results of mechanical stress from both ECM stiffness (Hernandez et al., 2010) and nuclear stiffness (Figure 6c). We have proposed the potential mechanisms of cytoskeletal stiffening in mediating nuclear blebbing, micronuclei formation, and cellular senescence, the involvement of the RhoA and Sun2 factors, and how increased ECM, nucleoskeletal, and cytoskeletal stiffness may be coupled in progeria cells.

Our results also demonstrate that there is sustained activation of RhoA signaling in progeria cells, which promotes F‐actin cytoskeletal stiffness and impairs proper F‐actin dynamics. RhoA activation in progeria cells could be a result of Sun2 activation as well as increased pro‐inflammatory signaling, pro‐fibrotic signaling, DNA damage, ROS production, and activated mechano‐sensing signaling (Li et al., 2011). Lamin A or Zmpste24‐deficiency has been shown to cause dysregulated differentiation capacity of stem cells, including increased osteogenic potential and repressed adipogenic potential. Our results strongly suggest that increased cytoskeletal stiffness and RhoA activation in progeria cells are determinant for this disrupted adipo‐osteogenic balance of cell fate.

Aging is associated with senescence and depletion of functional stem cells in various tissues, leading to impeded tissue homeostasis and regeneration capacity. Senescence and depletion of stem cells can be caused by both cell autonomous mechanisms and noncell autonomous mechanisms (Baar, Perdiguero, Munoz‐Canoves, & de Keizer, 2018). Senescent cells have a senescence‐associated secretory phenotype (SASP), which alters the microenvironment and impacts the function of nonsenescent cells (Baar et al., 2018). The SASP can affect the stem cell niche, impacting stem cell function via a noncell autonomous mechanism. In skeletal muscle, muscle stem cells (satellite cells) reside in niches at the basal lamina of myofibers and are responsible for the regeneration of skeletal muscle. Muscle stem cell behaviors are regulated by multiple microenvironmental cues/factors in and around the niches, such as myofibers, b*asal lamina,* extracellular matrix, neighboring nonmyogenic cells, and soluble factors secreted by other cells. Muscle stem cells are negatively impacted by pro‐inflammatory cytokines and pro‐fibrotic growth factors released by inflammatory cells, fibroblasts, and PDGFR‐α^+^ FAPs (or MSCs in the current study). Our previous studies of severely dystrophic muscle in mouse models have revealed that macrophage and MSC proliferation and function increased during disease progression in dystrophic muscles, whereas MPC proliferation and function declined (Mu et al., 2013; Sohn, Lu, Tang, Wang, & Huard, 2015). Similarly, homeostasis and regeneration of aged skeletal muscle can be impaired by macrophage‐mediated chronic inflammation and fibrogenic cell‐mediated fibrosis formation. SASP factors from senescent cells also can chronically impair muscle stem cell function and attenuate tissue regeneration in muscle. Therefore, the defective muscle regenerative capacity of muscle stem cells depends intimately on the microenvironment modulated by various types of senescent cells, and senescent *Z24* ^−/−^ MSCs could contribute to the impaired function of muscle stem cells in *Z24* ^−/−^ muscle. It is notable that increased intrinsic defects including nuclear abnormalities, DNA damage, and cellular senescence also can be induced by *LMNA* mutation in Pax7^+^ muscle stem cells, and the paracrine effect by MSCs is not the only causal factor of muscle stem cell defects. Importantly, treating Z24^−/−^ mice with a RhoA inhibitor reduced senescence, suggesting that RhoA inhibitors represent a novel type of senotherapeutic.

The increased micronuclei formation and cytoplasmic DNA induced by DNA damage can activate cGAS‐Sting innate immune signaling and contribute to cellular senescence (Dou et al., 2017). The potential correlation of cGAS‐Sting signaling with mechanical properties of a cell has not been addressed before our study. Current results suggest that increased RhoA activation, cytoskeletal stiffness, and Sun2 expression in progeria cells are associated with increased generation of nuclear blebs/micronuclei and cytoplasmic DNA fragments, thus mediating the activation of cGAS‐Sting signaling and cellular senescence progression.

Taken together, our novel results demonstrate that increased cytoskeletal stiffness and RhoA/Sun2 signaling represents a novel mechanism for promoting aging and cellular senescence and therefore represents a novel therapeutic target for intervention in progeria diseases and for extending healthspan with natural aging. These results extend our understanding regarding how mutations in lamin A, a structural and mechano‐responsive protein in nuclear lamina network, changes the mechanical properties of nuclear and cytoskeleton and contributes to driving cellular senescence and thus aging and age‐related diseases.

## 4 METHODS

### 4.1 Animal models

*Zmpste24* ^−/−^ (*Z24* ^−/−^ *)* mice (B6.129SZmpste24tm1Sgy/Mmucd) were studied as an established model for HGPS. The aged‐matched littermates (*Zmpste24^+/+^*) mice born from same *Zmpste24 ± *parents were used as wild‐type (WT) controls. Both male and female mice were used for this study since both genders are susceptible to HGPS disease. All mice were housed and maintained in the Center for Laboratory Animal Medicine and Care (CLAMC) at UTHealth in accordance with established guidelines and protocols approved by the UTHealth Animal Welfare Committee.

### 4.2 Muscle cell isolation and culturing

Muscle‐derived mesenchymal stem/stromal cells (MSCs) and muscle stem/progenitor cells (MPCs) were isolated from the skeletal muscle of *Z24* ^−/−^ mice and WT mice (~5 months old, male and female) using the modified preplate technique, based on their adhering capacity to collagen‐coated surface/substrate. MSCs adhere quickly in hours, whereas MPCs continue floating in medium and only attached and start to grow days later. Cells were cultured in proliferation medium (DMEM supplemented with 20% fetal bovine serum (FBS]). WT and *Z24* ^−/−^ MSCs were generally always cultured in collagen type I (0.1%)‐coated plastic flasks or plates for all the experiments in this study.

### 4.3 Human fibroblasts from HGPS and normal patients

The fibroblasts from HGPS patients and normal humans were kindly shared from the Coriell Institute and were cultured in DMEM medium supplemented with 15% fetal bovine serum (FBS), 1X glutamax, and 1% penicillin–streptomycin. The HGPS fibroblasts cell strains include AG03513, AG06917, and AG08466, and normal fibroblasts include AG08468, AG08469, and AG08470. Living cells gotten from Coriell Institute were cultured to passage 3 to be compared in our studies.

### 4.4 Atomic force microscopy (AFM) testing of cell stiffness

Cells were fixed 4% paraformaldehyde, and the cytoplasm or nuclear stiffness was measured with AFM system at room temperature (Collum et al., 2017). The force curves measurements were performed with a Catalyst Bioscope System (Bruker Corporation, Billerica, MA). The AFM was equipped with an inverted light microscope (Olympus IX81) to track the position of cell and AFM probe. The AFM probe has a material of nonconductive silicon nitride, with the spring constant values of approximately 0.05 N/m (Bruker. Model: MLCT; cantilever: T:0.55 µm). The cantilever sensitivity was calibrated with the NanoScope software by measuring a force curve on a clean silicon wafer. Force curves were acquired at a ramp size of 10 µm and ramp rate of 1.03 Hz. Young's modulus, E, was calculated from obtained force curves based on the active curve of “extend” and fit mode of Sneddon (conical) using NanoScope analysis program from the Bruker Corporation. The *F* = 2π*E*1 − υ2tanαδ2 where *F* = force, E = Young's modulus, ν = Poisson's ratio, α = half‐angle of the indenter, and δ = indentation depth (Collum et al., 2017).

### 4.5 Telomere staining

Telomeres in the interphase nuclei were detected using a Cy3‐conjugated peptide nucleic acid (PNA) probe provided with the Telomere PNA FISH Kit (Agilent). Fluorescence in situ hybridization of telomere was performed according to the manufacturer's instruction. Briefly, the cells were heated at 80°C for 5 min to denature DNA in the presence of the Cy3‐conjugated PNA probe followed by hybridization in the dark at room temperature (RT) for 30 min. The hybridization was followed by a brief rinse with a Rinse Solution, and a posthybridization wash with a Wash Solution at 65°C for 5 min. DAPI was used to counterstain DNA.

### 4.6 Treatment of *Z24* ^−/−^ MSCs with F‐actin stabilizing and destabilizing effectors

*Z24* ^−/−^ MSCs cultured in collagen‐coated plastic plates were treated with growth medium supplemented with Jasplakinolide (JPK) (200 nM) to induce F‐actin stabilization or with Cytochalasin D (CyD) (100 ng/ml) to induce F‐actin depolymerization for 48 hr. The effect of F‐actin modification on nuclear blebbing was compared by immune‐staining of lamin A/C or Sun2.

### 4.7 In vitro RhoA inhibition assays

*Z24* ^−/−^ MSCs or HGPS fibroblasts cultured in collagen‐coated plastic plates were treated with a specific RhoA/Rho kinase (ROCK) inhibitor Y‐27632 (EMD Millipore, Billerica, MA) (10 μM) for 48 hr in cell proliferation medium, and the potential changes of F‐actin polymerization, nuclear blebbing, lamin A/C, Sun2, γ‐H2AX, and Histone methylation/acetylation markers were observed. RhoA‐specific inhibition also was performed with C3 transferase (5 µg/ml) treatment for 48 hr to verify the effect of RhoA inhibition on F‐actin polymerization and nuclear blebbing.

### 4.8 Measurement of cellular senescence

The percent of senescent muscle cells cultured in vitro (10% FBS in DMEM for 4 days) and in skeletal muscle tissue was measured using the senescence‐associated β‐Galactosidase (SA‐β‐gal) Staining Kit (Cell Signaling Technology) following the manufacturer's protocol. The number of cells positive for β‐gal activity at pH 6, a known characteristic of senescent cells, was determined.

### 4.9 Preparation of conditioned medium (CM)

The WT MSCs and *Z24* ^−/−^ MSCs were plated and grown to ~90% confluence in proliferation medium, and the culture medium was changed to low‐serum medium (DMEM with 2% FBS) to initiate the conditioning of culture medium. The cells were continued to be cultured for 48 hr to collect the CM by filtering through 0.4‐µm filter. The cell number of WT MSCs and *Z24* ^−/−^ MSCs was similar when CM was initiated and collected. The CM was mixed with same volume of fresh medium (1:1) when being used for cell treatment.

### 4.10 In vitro myogenic, osteogenic, and adipogenic differentiation assays

MPCs from 5‐month‐old WT or Z24^−/−^ mice (30 000 cells per well of 12‐well collage type I‐coated plastic plate), maintained in DM, 2% horse serum in DMEM (Invitrogen), were cultured with different conditioned medium (CM) from *Z24* ^−/−^ MSCs with or without Y‐27632 pretreatment. The *Z24* ^−/−^ MSCs were pretreated with or without Y‐27632 for 48 hr and continued to be cultured for another 48 hr to harvest the CM. Z24^−/−^ MPCs were then treated with the CM for 4 days to observe the progression of myogenic differentiation. The number of myotubes was determined by immunocytochemical staining with a fast‐type myosin heavy chain (f‐MHC) antibody (Sigma‐Aldrich). Osteogenic differentiation was performed by culturing the Z24^−/−^ MSCs in 12‐well plate in osteogenic differentiation medium which contains DMEM, 10% FBS, supplemented with dexamethasone (0.1 µm, Sigma‐Aldrich), ascorbic‐acid‐2‐phosphate (50 µg/ml, Sigma‐Aldrich), and 10 mm β‐glycerophosphate (Sigma‐Aldrich) and BMP2 (100 ng/ml, Medtronic). Cells were cultured in osteogenic differentiation medium for 4 days, and osteogenesis was assessed using a Fast Blue Alkaline Phosphatase (ALP) Kit (Sigma‐Aldrich). Adipogenic differentiation assay was performed with *Z24* ^−/−^ MSCs in adipogenic differentiation medium (Lonza). When the cells reached 100% confluence, three cycles of induction/maintenance medium were applied to the cells to induce optimal adipogenic differentiation. Adipogenesis was assessed using AdipoRed reagent (30 µl/ml, Thermo Fisher) and 4′,6‐diamidino‐2‐phenylindole (DAPI).

### 4.11 Constant activation of RhoA in MSCs

Rho activator II (Cytoskeleton Inc.), which can robustly increase the level of GTP‐bound RhoA and result in constitutively active Rho (Schmidt et al., 1997), was added to WT MSCs cultured on collagen‐coated plates at 200 ng/ml for 24 hr to observe increased RhoA protein and F‐actin polymerization. Expression of constitutively active RhoA‐GFP was also performed with amplified plasmid of pcDNA3‐EGFP‐RhoA‐Q63L (Addgene, plasmid #12968). Expression of a dominantly negative RhoA‐GFP with amplified plasmid of pcDNA3‐EGFP‐RhoA‐T19N (Addgene, plasmid #12967) served as a negative control. Lipofectamine 3,000 (Thermo Fisher) was applied to facilitate the plasmid transfection into WT MSCs.

### 4.12 Repression of Sun2 expression in Z24^−/−^ MSCs with siRNA

Z24^−/−^ MSCs cultured in plates were transfected with siRNA of Sun2 gene (Ambion siRNA from Thermo Fisher; AM16708, Sun2 SiRNA/mouse) (sense sequence: GCAUCACCAAGACUCGGAATT; anti‐sense sequence: UUCCGAGUCUUGGUGAUGCTC) to repress Sun2 expression, and cells were fixed for observation 48 hr posttransfection. Cells transfected with a control SiRNA served as control cells.

### 4.13 Epigenetics assay

The heterochromatin markers histone H3K9me3 and H3K27me3 were immunostained in *Z24* ^−/−^ MSCs with and without Y‐27632 treatment (10 μM for 48 hr) or Sun2 siRNA treatment, and HGPS cells with and without Y‐27632 treatment. The anti‐H3K9me3 and H3K27me3 antibodies were from Abcam.

### 4.14 RhoA and ROCK activity assays

Relative RhoA activity was measured in WT MSCs, *Z24* ^−/−^ MSCs, Y‐27632‐treated *Z24* ^−/−^ MSCs, and Sun2 siRNA‐treated *Z24* ^−/−^ MSCs to verify the activation state of RhoA, with a RhoA G‐LISA Activation Assay kit (Cytoskeleton Inc.), according to the manufacturer's instructions. Briefly, cells were lysed and snap‐frozen in liquid nitrogen. Approximately 30–40 μg total protein was used for each sample. The active GTP‐bound form of RhoA was detected with a specific anti‐RhoA antibody. Absorbance readings were obtained by measuring optical density (*OD*) at 490 nm. Relative ROCK activity was measured in *Z24* ^−/−^ MSCs and Y‐27632‐treated *Z24* ^−/−^ MSCs to verify the specificity of RhoA/ROCK inhibition, with a ROCK Activity Assay kit (Cell Biolabs, *San Diego*, CA), according to the manufacturer's instructions. The level of ROCK *activation* was determined by measuring *OD* at 450 nm.

### 4.15 qRT‐PCR

Total RNA was obtained from muscle cells or muscle tissues using the RNeasy Mini Kit (Qiagen, Inc.). Reverse transcription was performed using an iScript cDNA Synthesis Kit (Bio‐Rad Laboratories, Inc.). The sequences of primers were given in Table S1 for SASP genes (IL‐1α, IL‐1β, IL‐6, TNF‐α, TNFR1, MCP1, and CxCl2), IL‐10, Klotho, PDGFRs, GAPDH (glyceraldehyde 3‐phosphate dehydrogenase), etc.. PCR reactions were performed using an iCycler thermal cycler (Bio‐Rad Laboratories, Inc.). The cycling parameters used for all primers were as follows: 95°C for 10 min; PCR, 40 cycles of 30 s at 95°C for denaturation, 1 min at 54–58°C for annealing, and 30 s at 72°C for extension. Products were separated by size and visualized on a 1.5% agarose gel stained with ethidium bromide. All data were normalized to the expression of GAPDH.

### 4.16 In vivo RhoA inhibition with Y‐27632

Systemic inhibition of RhoA/ROCK signaling was conducted by i.p. (Intraperitoneal) injection of 10 mg/kg Y‐27632 (5 mM in phosphate‐buffered saline [PBS]) into *Z24* ^−/−^ mice, starting at 10 weeks of age with PBS used as the vehicle control. The i.p. injections of Y‐27632 into *Z24* ^−/−^ mice were conducted three times a week for 12 weeks.

### 4.17 Cardiotoxin‐induced muscle injury

In order to compare the muscle regeneration potential in WT mice, *Z24* ^−/−^ mice, and *Z24* ^−/−^ mice treated with Y‐27632, cardiotoxin (Sigma) (10 μM in 40 μl of PBS) was injected intramuscularly into the GM muscles of mice (~20‐week old) to induce muscle injury. Muscle tissues were collected at 7 days after the injection of cardiotoxin to compare fibrosis formation, muscle regeneration, and number of senescent cells.

### 4.18 Histology and immunofluorescent staining

Frozen tissue sections were fixed with 10% formalin, and cultured cells were fixed with 4% paraformaldehyde. All of the primary antibodies—lamin A/C (Santa Cruz), Pax7 (DHSB), CD68 (Abcam), PDGFR‐α (Abcam), fast‐type myosin heavy chain (f‐MHC) (Abcam), Sun1 (Novus Biologicals), Sun2 (Abcam), p21 (Cell signaling), γ‐H2AX (Cell Signaling), RhoA (Santa Cruz and Abcam), H3K9me3 (Abcam), and H3K27me3 (Abcam)—were used at a 1:100 to 1:300 dilution. All slides were analyzed via fluorescence microscopy (Nikon Instruments Inc.) and photographed at 4–40 × magnification. F‐actin was stained with Alexa Fluor 488 Phalloidin or Alexa Fluor 594 Phalloidin (Thermo Fisher). G‐actin was detected with an antibody to DNase I which specifically bind to G‐actin (Abcam). The cell nuclei were stained with DAPI. Fibrosis formation in muscle tissues was visualized by Masson trichrome staining with the Trichrome Stain (Masson) Kit (Sigma‐Aldrich). Sections were incubated in Weigert's iron hematoxylin working solution for 10 min and rinsed under running water for 10 min. Slides were transferred to Biebrich scarlet‐acid fuchsin solution for 15 min before incubation in aniline blue solution for another 5 min. Slides were then rinsed, dehydrated, and mounted as earlier. The ratio of the area of fibrotic collagen (blue) to the area of normal muscle (red) was quantified to measure fibrosis formation.

### 4.19 Measurements of results and statistical analysis

Image analysis was performed using Nikon *NIS‐Elements* (Nikon Instruments Inc.) and Image J software (National Institutes of Health). Data from at least three samples were pooled for statistical analysis. Results are given as the mean ± standard deviation (*SD*). The statistical significance of any difference was calculated using Student's *t* test or one‐way ANOVA test. *P* values < .05 were considered statistically significant.

## CONFLICT OF INTEREST

Johnny Huard discloses the fact that he receives royalties from Cook Myosite, Inc. for muscle stem cell technologies.

## AUTHOR CONTRIBUTIONS

XM, PR, and JH designed research. XM, CT, WH, CL, PC, PM, WC, KS, JG, PG, SR, EM, YC, and LZ performed the experiments. PC, PR, LN, and JC contributed drugs, protocols, and critique. XM, CT, WH, WC, JG, and LZ analyzed the data. XM, PR, LN, and JH wrote the manuscript.

## Supporting information

Supplementary Material

## Data Availability

The data that support the findings of this study are available from the corresponding author upon reasonable request.
