# Supplementary material for: Cytoskeleton stiffness regulates cellular senescence and innate immune response in Hutchinson–Gilford Progeria Syndrome
Source: Aging Cell. 2020 Jul 25;19(8):e13152. doi: 10.1111/acel.13152 (PMC7431831; doi:10.1111/acel.13152)
Supplement: Supplementary file 1 — Supplementary Material [file ACEL-19-e13152-s001.docx]

**
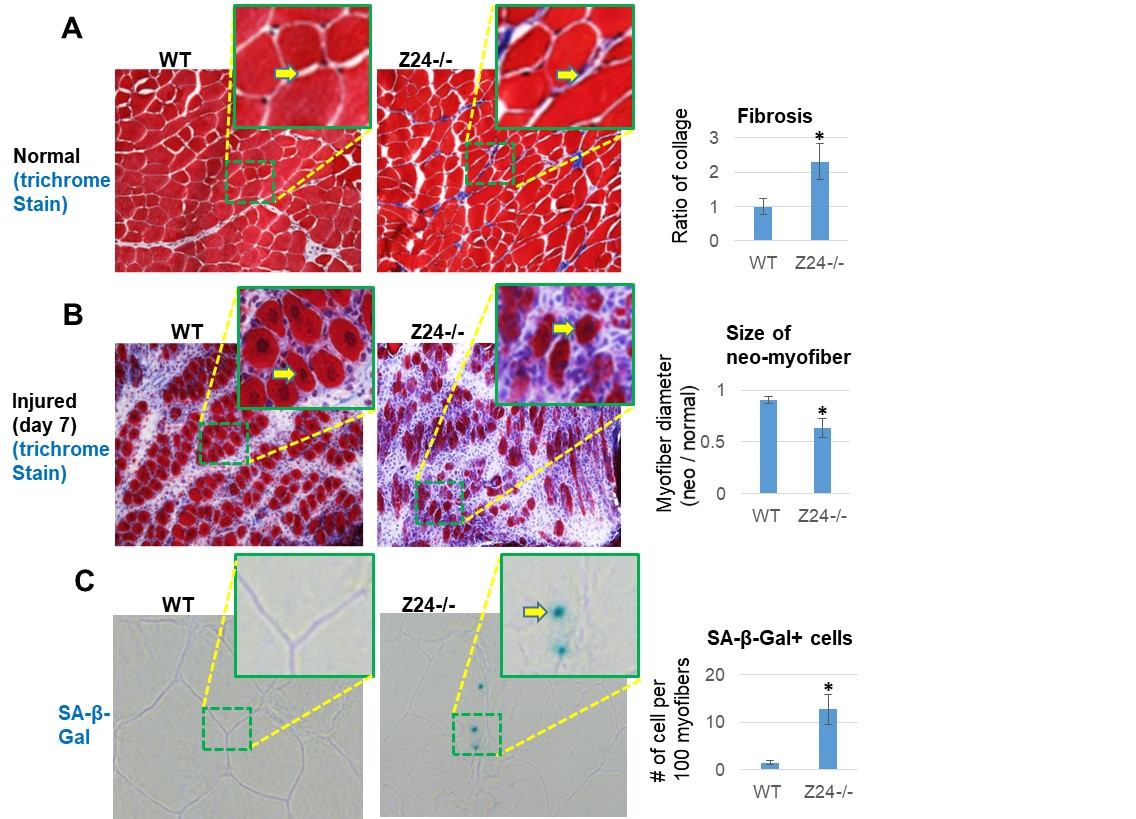
**

**Supplemental Figure 1. Increased senescent phenotypes in the skeletal muscle of *Z24*^-/-^ mice.**

**A.** Trichrome staining of muscle tissues of WT and *Z24^-/-^* mice to examine fibrosis formation. Quantification of fibrosis (collagen deposition) is shown. Arrow: collagen (blue). **B**. Trichrome staining of regenerating muscle tissues 7 days after cardiotoxin-induced injury. Quantification of the size of regenerating neo-myofibers (positive with centrally located nuclei) is shown. Arrow: neo-myofiber with centrally located nuclei. **C.** SA-β-Gal staining of muscle tissues of WT and *Z24^-/-^* mice to examine to examine senescent cells. Quantification of SA-β-Gal+ cells. N>=6. “*” at bar charts indicates p<0.05.

**
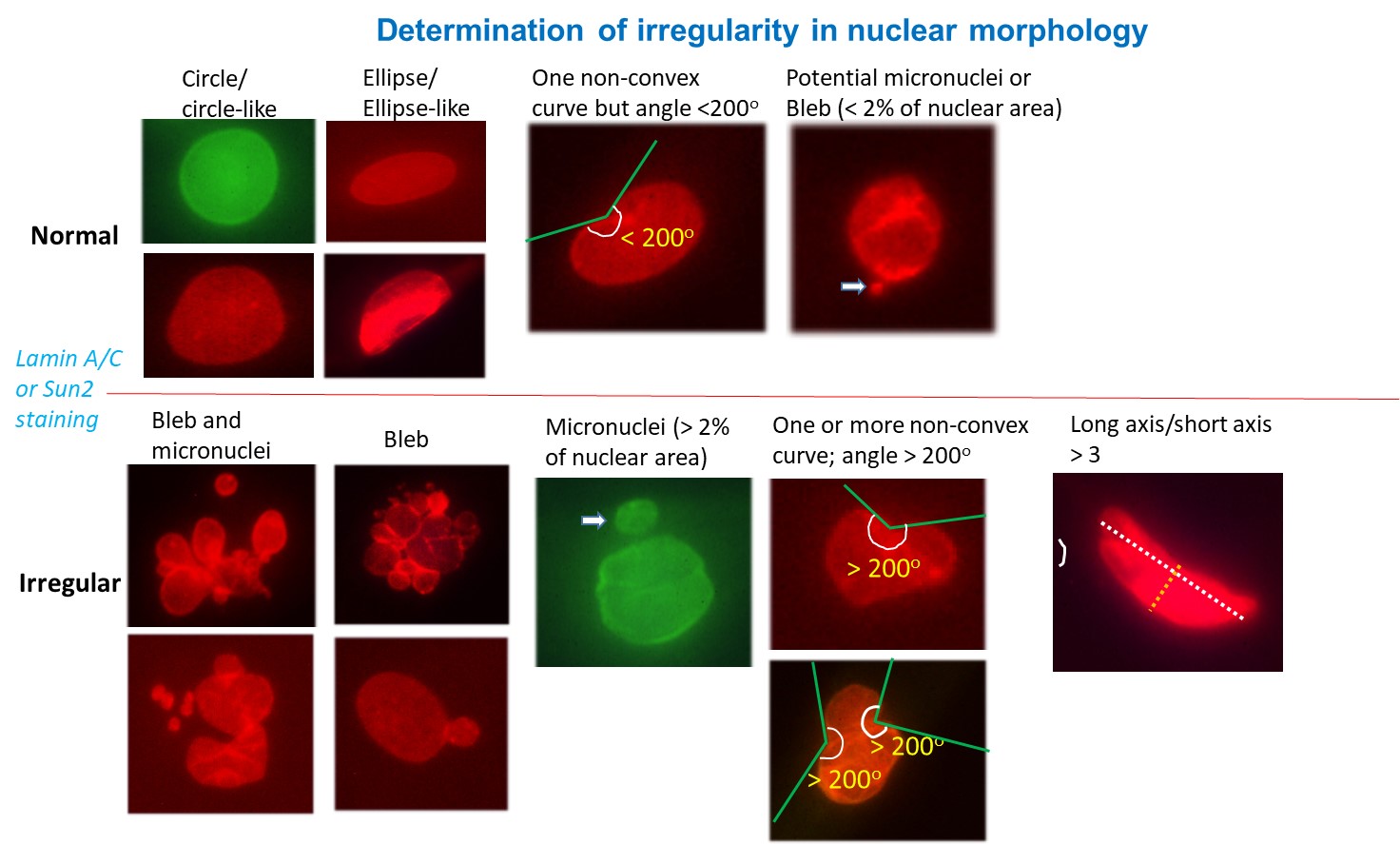
**

**Supplemental Figure 2. The standard settings for determining the irregularity of nuclear morphology.**

The normal nucleus and irregular nucleus are generally classified with these settings. The statistics of the nuclear blebbing or irregularity in cells is based on this setting.


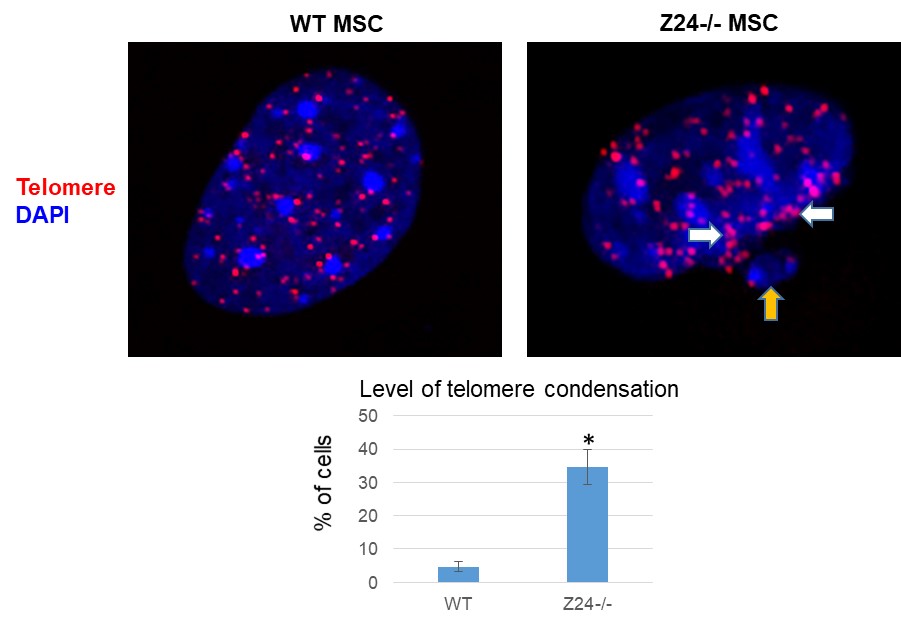


**Supplemental Figure 3. Increased telomere dislocation and condensation in Z24^-/-^ MSCs.**

Cy3-telomere probe analysis of WT MSC and *Z24^-/-^* MSC. Orange arrow shows translocated telomere into micronuclei. White arrow shows increased accumulation/condensation of telomere signal in the nucleus. Quantification of telomere condensation is shown. N>=6. “*” at bar charts indicates p<0.05.


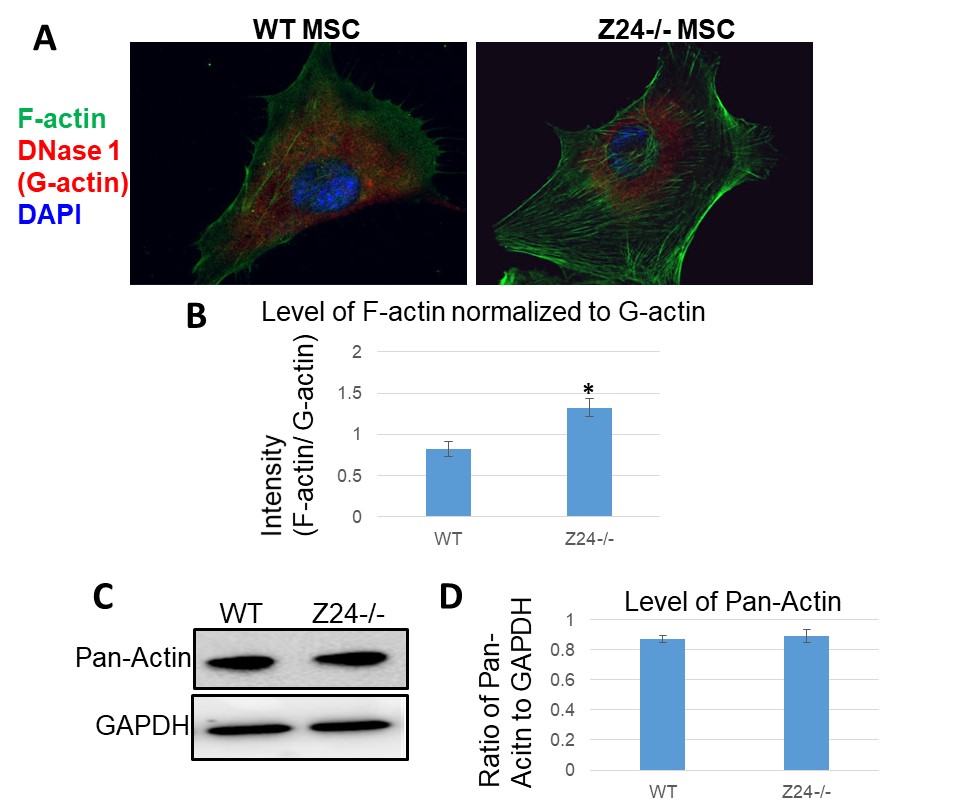


**Supplemental Figure 4. F-actin level but not total actin level is increased in *Z24^-/-^* MSCs.**

**A.** Immunostaining of DNAase I (specifically binds to G-actin) and F-actin in WT MSC and *Z24^-/-^* MSC. B. Quantification of F-actin level normalized to G-actin level is shown. **C**. Western blot analysis of pan-actin level in WT MSCs and *Z24^-/-^* MSCs. **D.** Quantification of pan-actin (total actin) level in western blot result is shown. N>=3. “*” at bar charts indicates p<0.05.

**
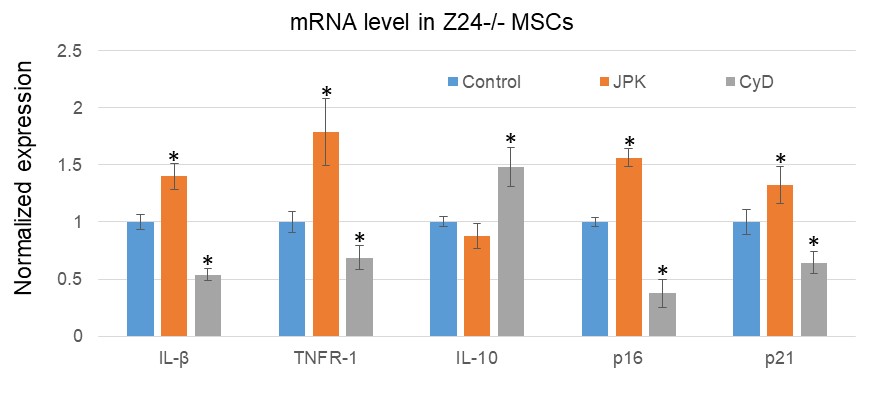
**

**Supplemental Figure 5. Increased expression of senescence related factors in *Z24*^-/-^ MSCs.**

qPCR analysis of the expression of senescence related factor (IL-1β, TNFR1, TL-10, p16 and p21) in WT MSCs and *Z24*^-/-^ MSCs. Expression levels were normalized to GAPDH. N>=3. “*” at bar charts indicates p<0.05.

**
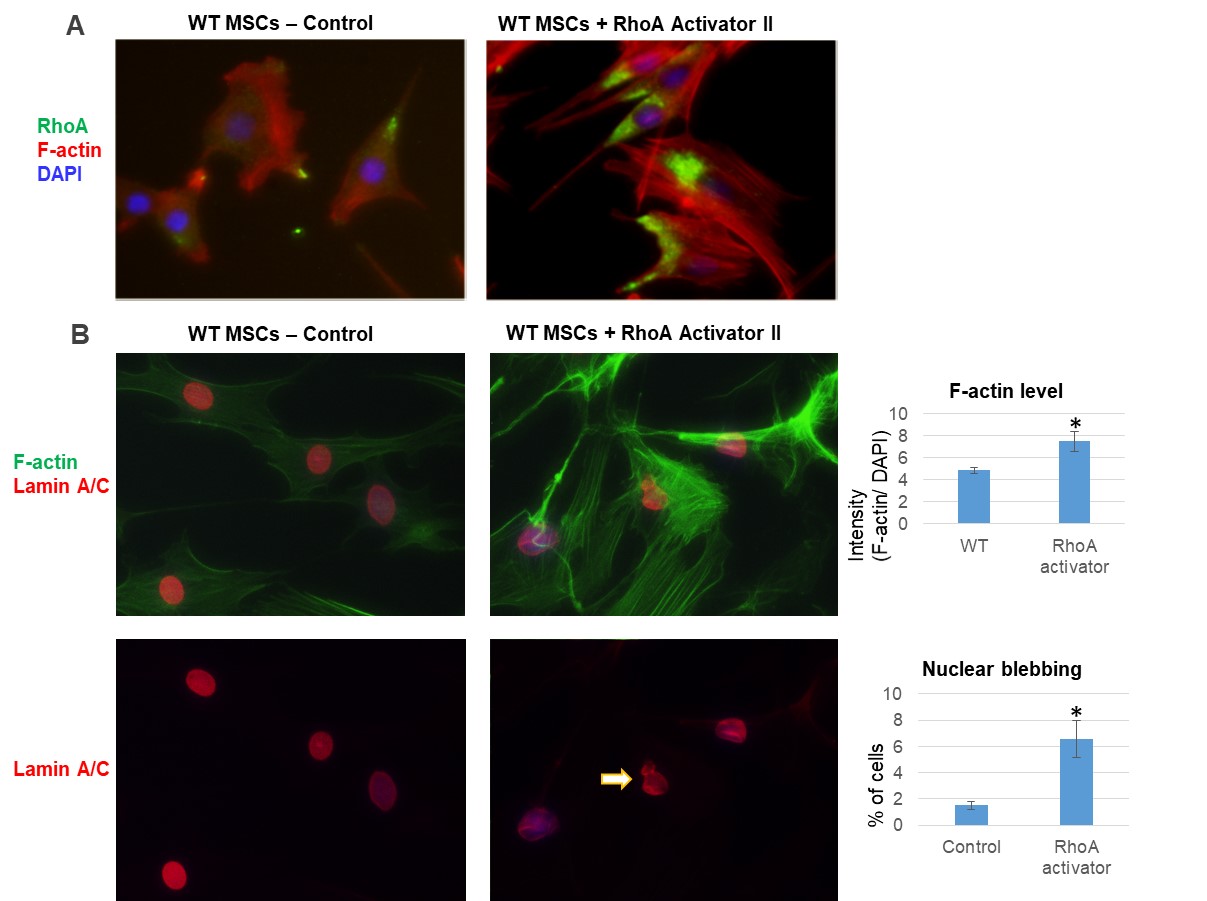
**

**Supplemental Figure 6. Activation of RhoA in WT MSCs by RhoA activator leads to increased F-actin polymerization and nuclear blebbing.**

**A.** Immunostaining analysis of RhoA and F-actin in WT MSCs treated with Rho Activator II from Cytoskeleton Inc. for 24 hr. **B.** Immunostaining analysis of Lamin A/C and F-actin in WT MSCs treated with the Rho activator II. Quantification of F-actin and nuclear blebbing is shown. N>=6. “*” at bar charts indicates p<0.05.


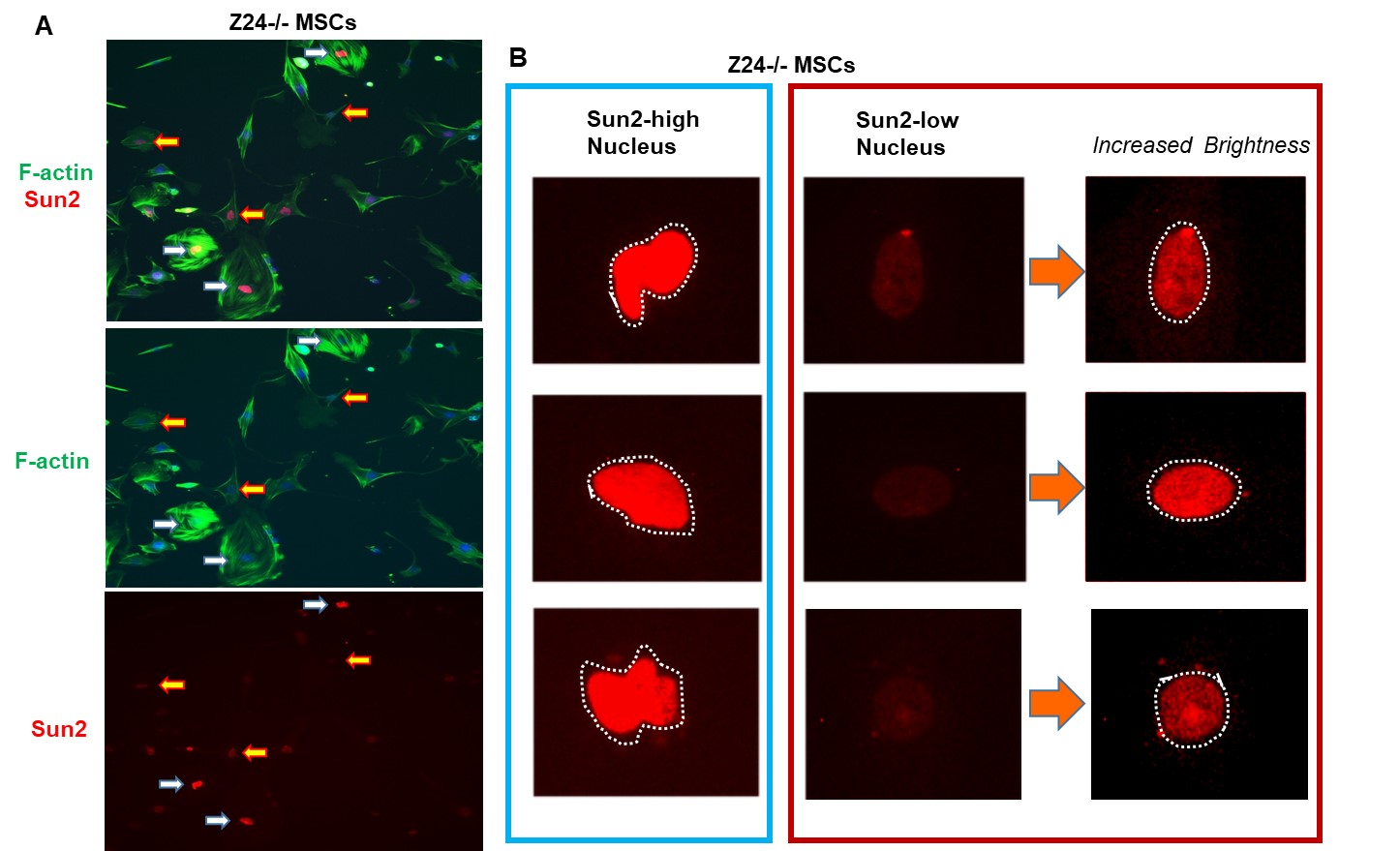


**Supplemental Figure 7. Elevated Sun2 expression is closely associated with increased F-actin and nuclear blebbing.**

**A.** Immunostaining analysis of Sun2 and F-actin in *Z24*^-/-^ MSCs. Yellow arrows: cells with lower level of Sun2 protein and F-actin; white arrows: cells with higher level of Sun2 protein and F-actin. **B.** The morphology of single cell nuclei with highest and lowest Sun2 expression are shown.

**
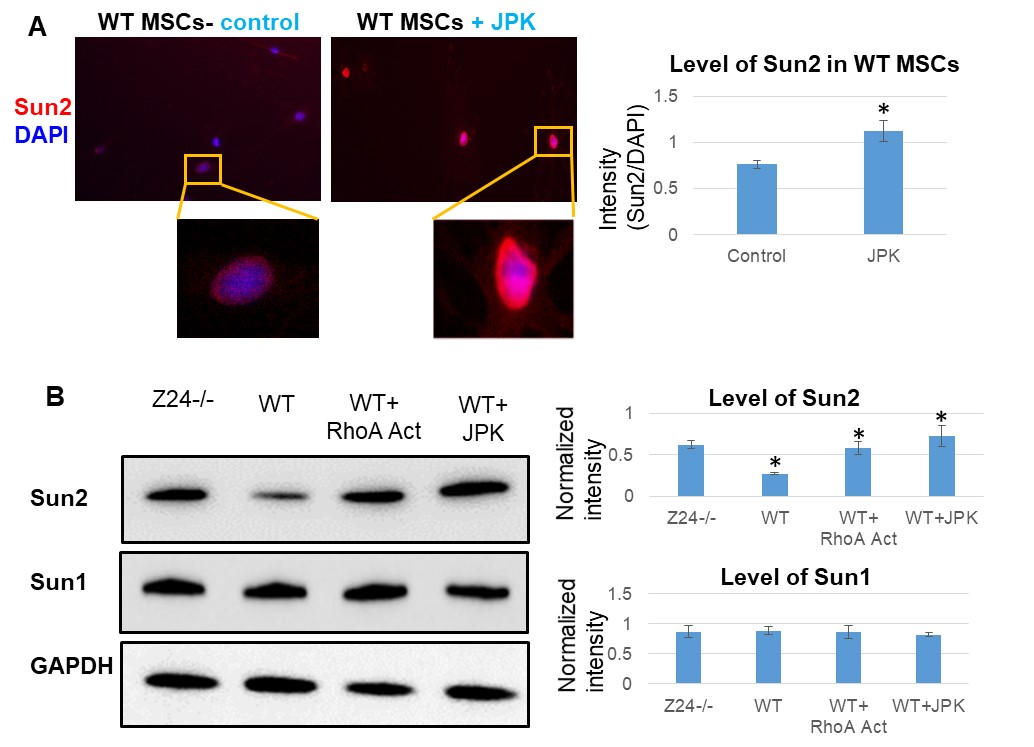
**

**Supplemental Figure 8. Effect of JPK treatment on the expression of Sun proteins in WT MSCs.**

**A.** Immunostaining analysis of Sun2 in WT MSC with or without JPK treatment. Quantification of the level of Sun2 is shown. **B.** Western blot analysis of Sun1 and Sun2 in *Z24*^-/-^ MSCs, WT MSCs, and WT MSCs treated with RhoA activator or JPK. Quantification of the level of Sun1 and Sun2 is shown. N>=6. “*” at bar charts indicates p<0.05.

**
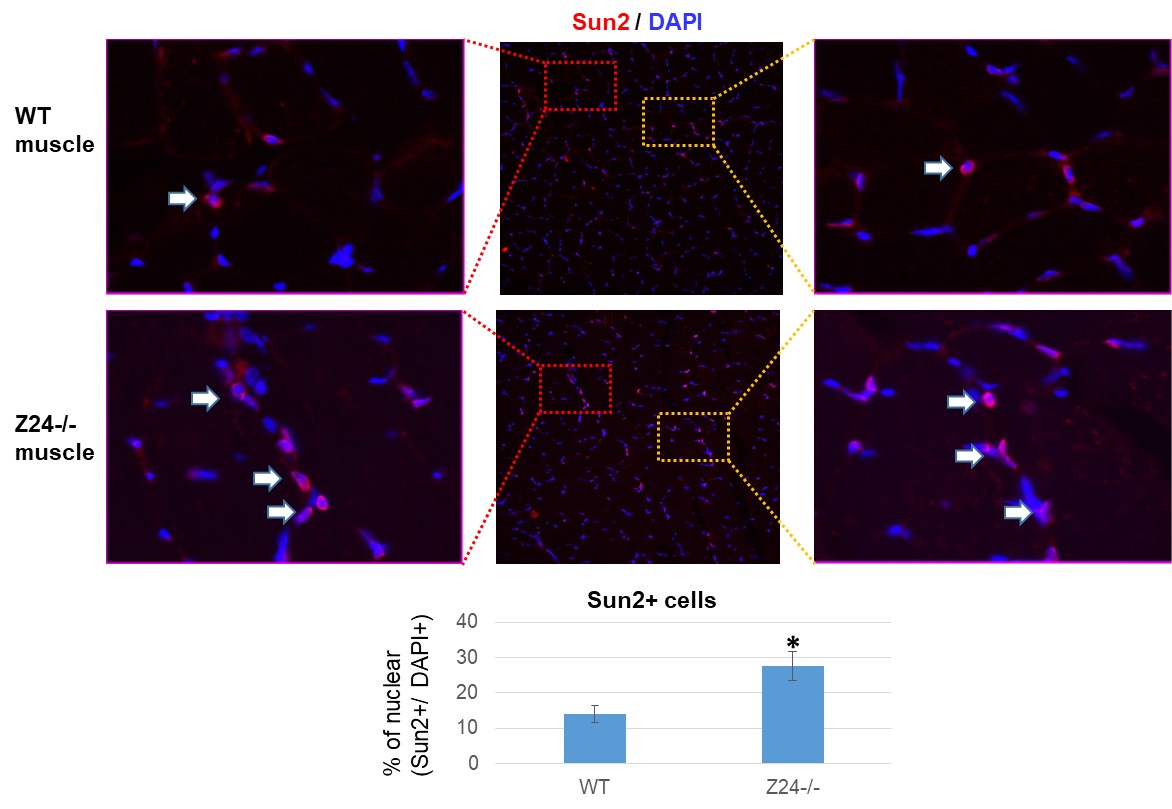
**

**Supplemental Figure 9. Increased Sun2 expression in the skeletal muscle of *Z24*^-/-^ mice.**

Immunohistochemical analysis of Sun2 and DAPI in the skeletal muscle from 5-month old WT or *Z24*^-/-^ mice. White arrows indicate the Sun2+ cells in the *Z24*^-/-^ muscle. Quantification of Sun2+ cells in muscles is shown. N>=6. “*” at bar charts indicates p<0.05.

**
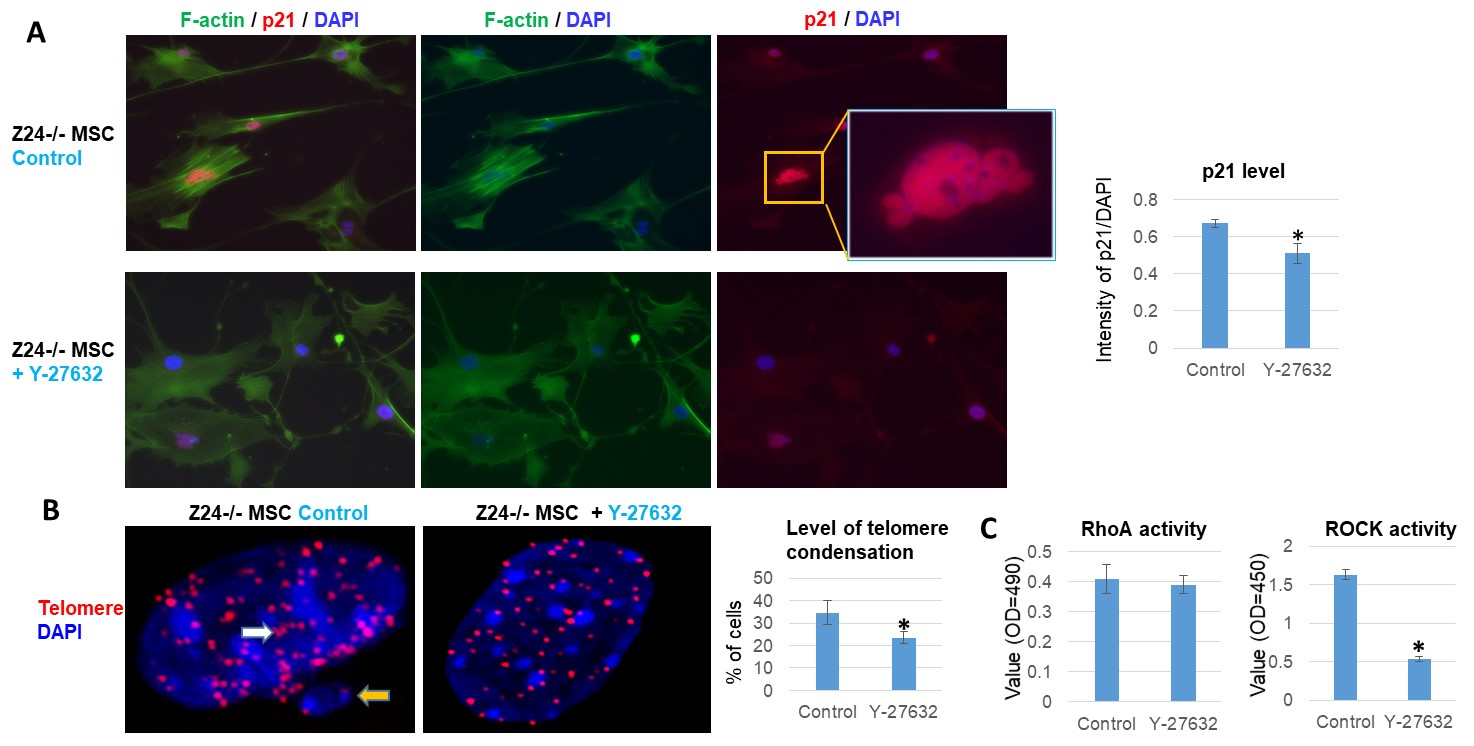
**

**Supplemental Figure 10. RhoA inhibition reduced p21**^Cip1^ **expression and telomere dislocation in *Z24*^-/-^ MSCs.**

**A.** Immunostaining analysis of p21^Cip1^ and F-actin in *Z24*^-/-^ MSCs with or without Y-27632 treatment. Quantification of p21 level is shown. **B.** Cy3-telomere probe staining to detect translocation of telomere into micronuclei (orange arrow), and accumulation/condensation of telomeres in the nucleus (white arrows). Quantification of the level of telomere condensation is shown. **C.** Quantification of RhoA and ROCK activity in *Z24*^-/-^ MSCs with or without Y-27632 treatment is shown. N>=6. “*” at bar charts indicates p<0.05.

**
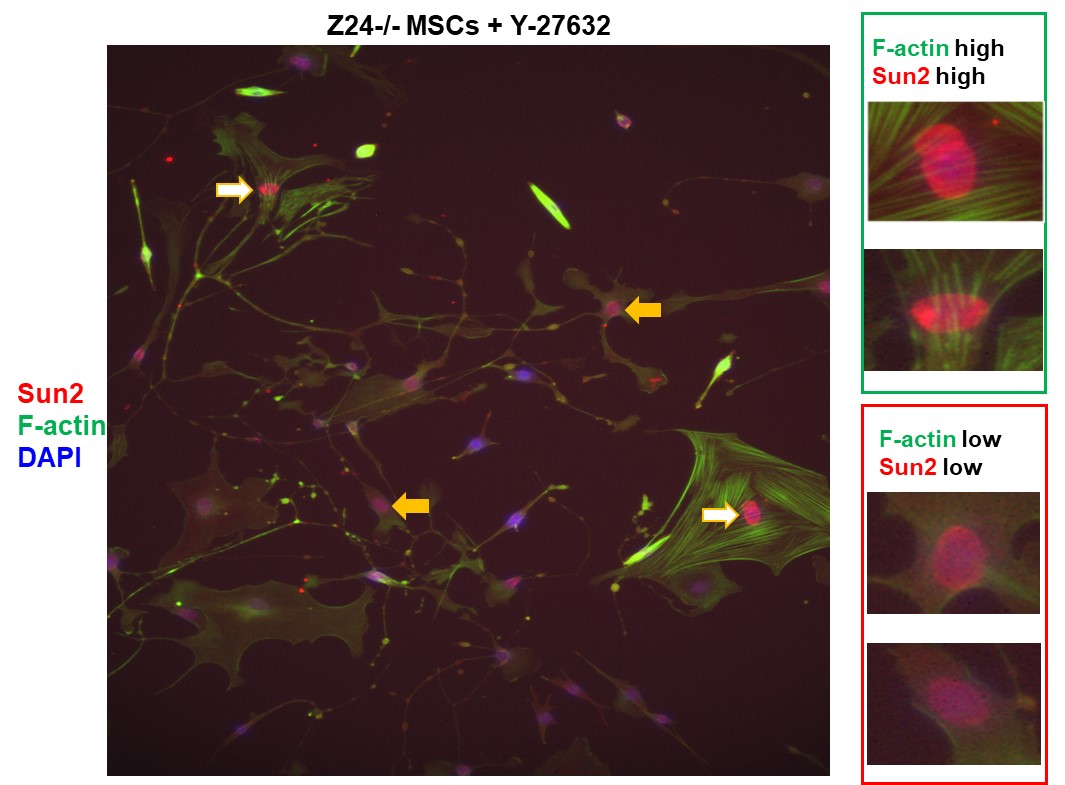
**

**Supplemental Figure 11. Depolymerization of F-actin is coupled with decreased Sun2 expression in Y-27632 treated *Z24^-/-^* MSCs.**

Immunostaining analysis of Sun2 and F-actin in Y-27632-treated *Z24*^-/-^ MSCs. White arrow indicate cells with higher level of F-actin and Sun2. Orange arrow indicate cells with lower level of F-actin and Sun2.

**
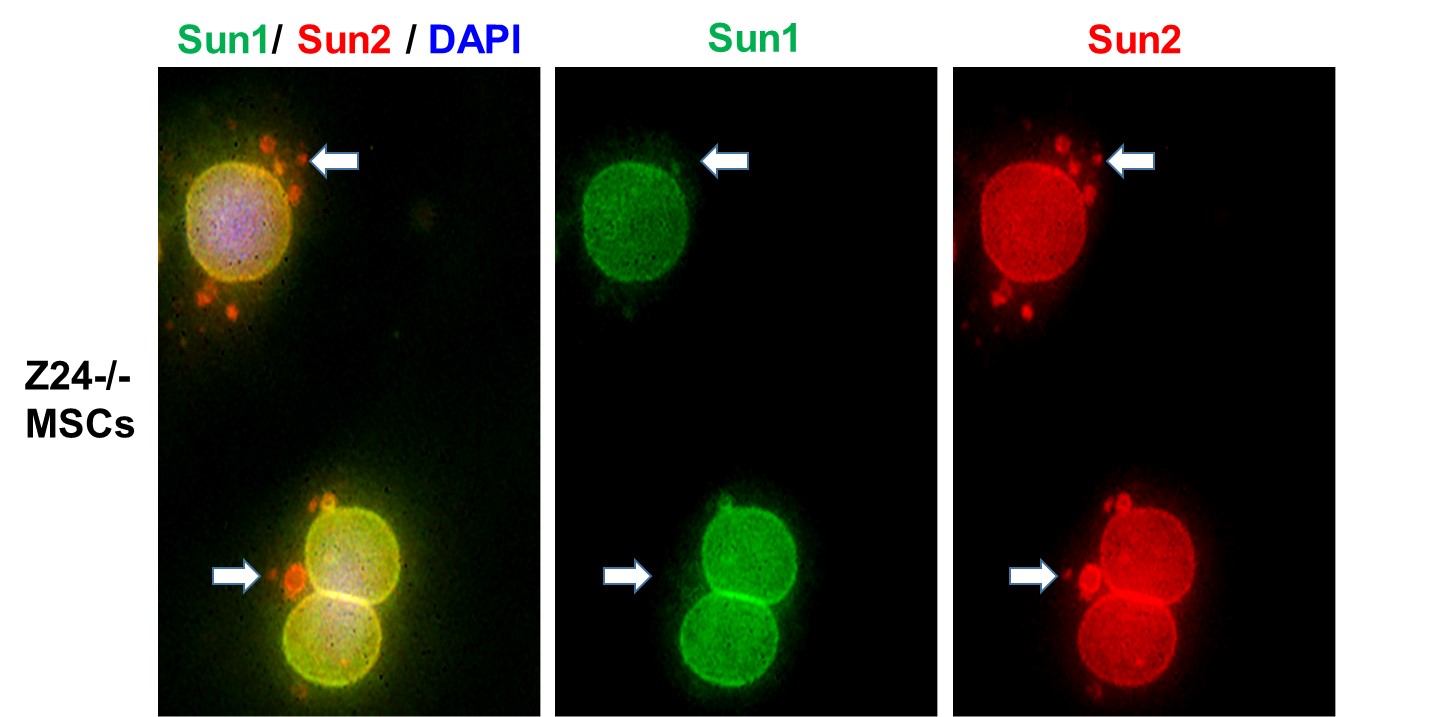
**

**Supplemental Figure 12. Sun2 but not Sun1 is more specifically activated in micronuclei of *Z24*^-/-^ MSCs.**

Immunostaining analysis of Sun1 (green) and Sun2 (red) in *Z24*^-/-^ MSCs. Arrows: micronuclei.


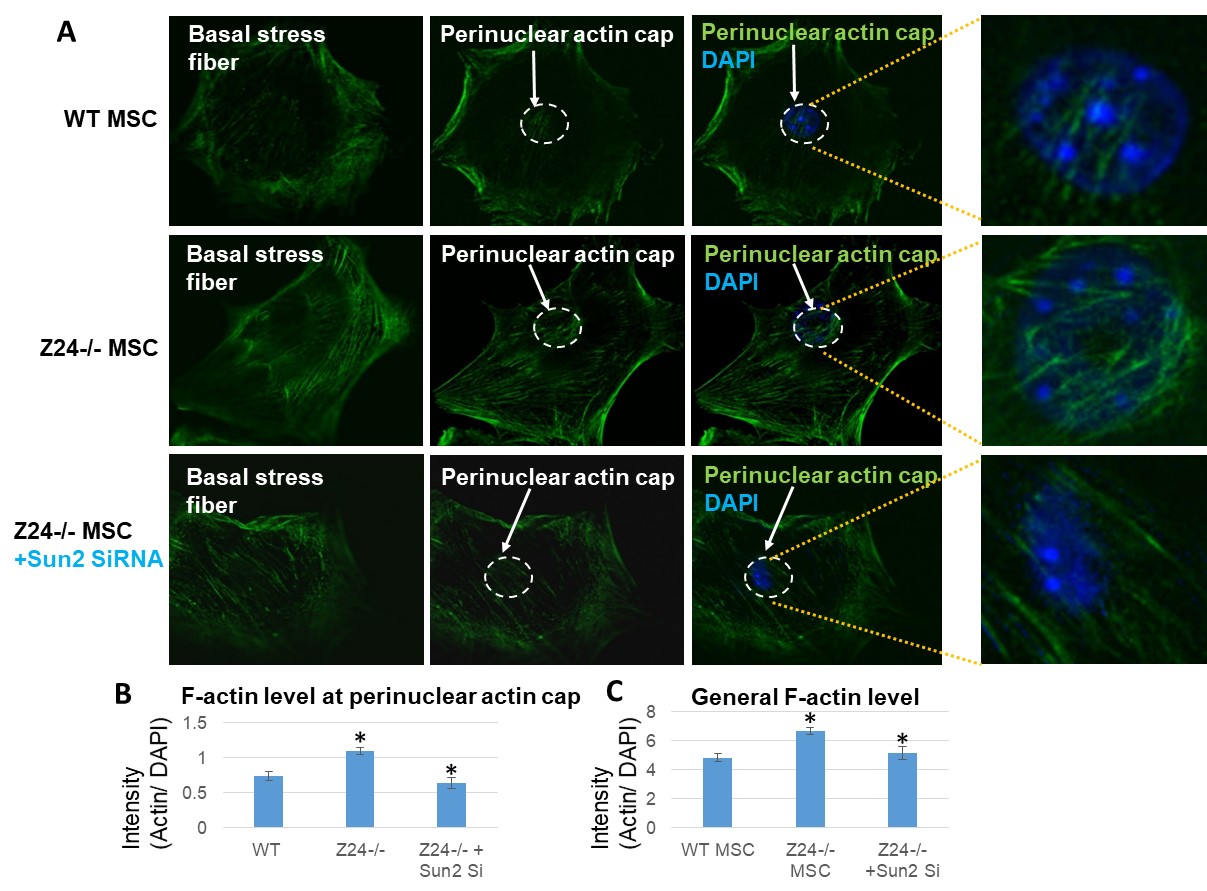


**Supplemental Figure 13. Repression of Sun2 decreased F-actin level at perinuclear actin cap of *Z24*^-/-^ MSCs.**

**A.** Phalloidin staining of F-actin (green) reveals the differential level of F-actin at perinuclear actin cap location in WT MSCs, *Z24*^-/-^ MSCs and *Z24*^-/-^ MSC treated with Sun2 SiRNA. **B.** Quantification of F-actin level at perinuclear actin cap is shown. **C.** Quantification of general F-actin level in the cell is shown. N>=3. “*” at bar charts indicates p<0.05.


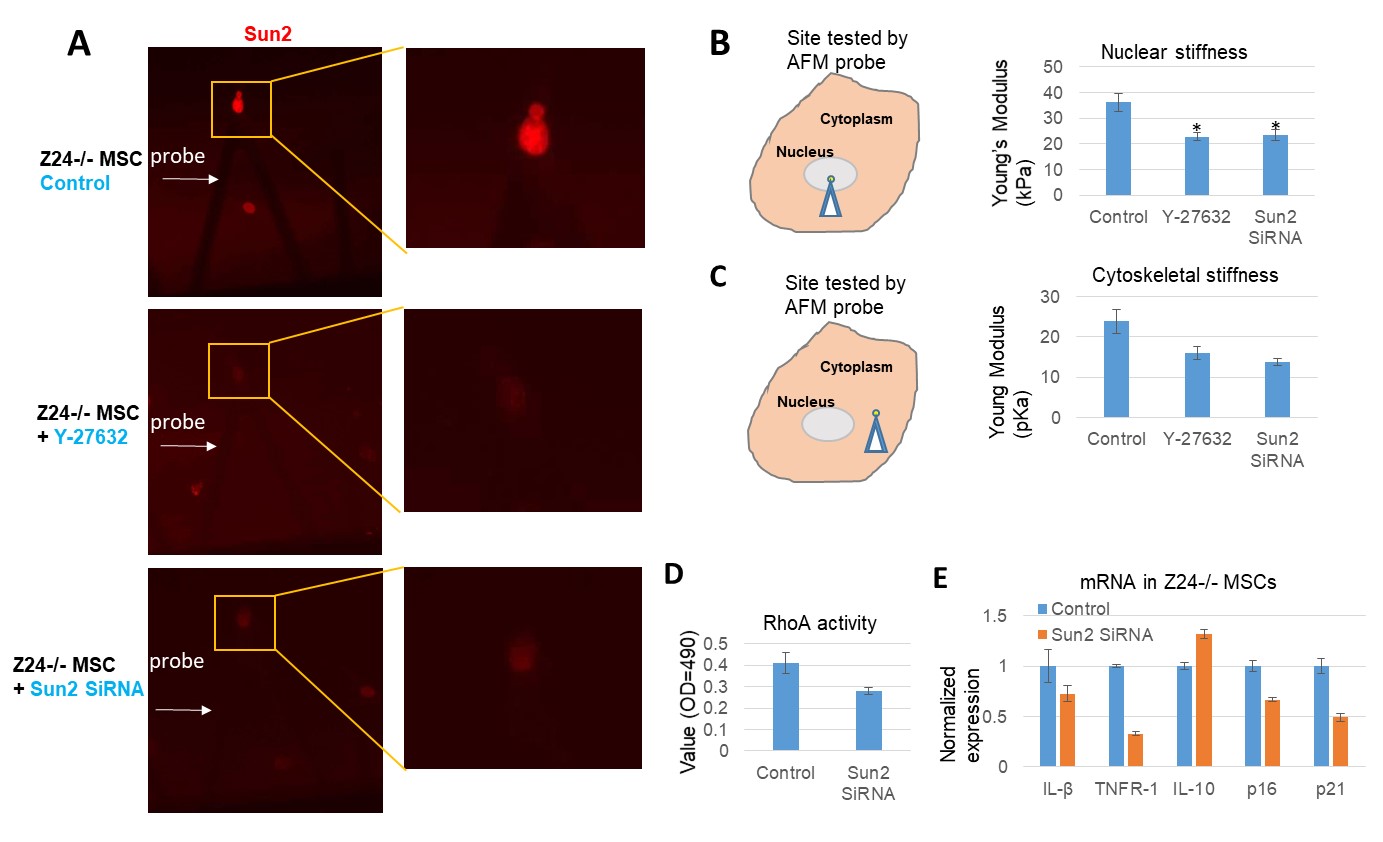


**Supplemental Figure 14. Cell stiffness, RhoA activity and expression of senescence genes were decreased in *Z24*^-/-^ MSCs treated with Sun2 SiRNA or Y-27632.**

**A.** AFM probe testing of the stiffness of *Z24^-/-^* MSCs and *Z24^-/-^* MSCs treated with Y-27632 or Sun2 SiRNA. Immunostaining analysis of Sun2 was performed to visualize/localize cell nucleus in the dish. **B.** AFM probe testing of cell stiffness at nuclear location. Quantification of cell stiffness with NanoScope analysis is shown. **C.** AFM probe testing of cell stiffness at cytoplasmic location. Quantification of cell stiffness with NanoScope analysis is shown. **D**. Quantification of RhoA activity in *Z24^-/-^* MSCs with or without Sun2 SiRNA treatment. **E.** qPCR analysis of the expression of senescence related factor (IL-1β, TNFR1, TL-10, p16 and p21) in *Z24*^-/-^ MSCs with or without Sun2 SiRNA treatment. Expression levels were normalized to GAPDH. N>=3. “*” at bar charts indicates p<0.05.

**
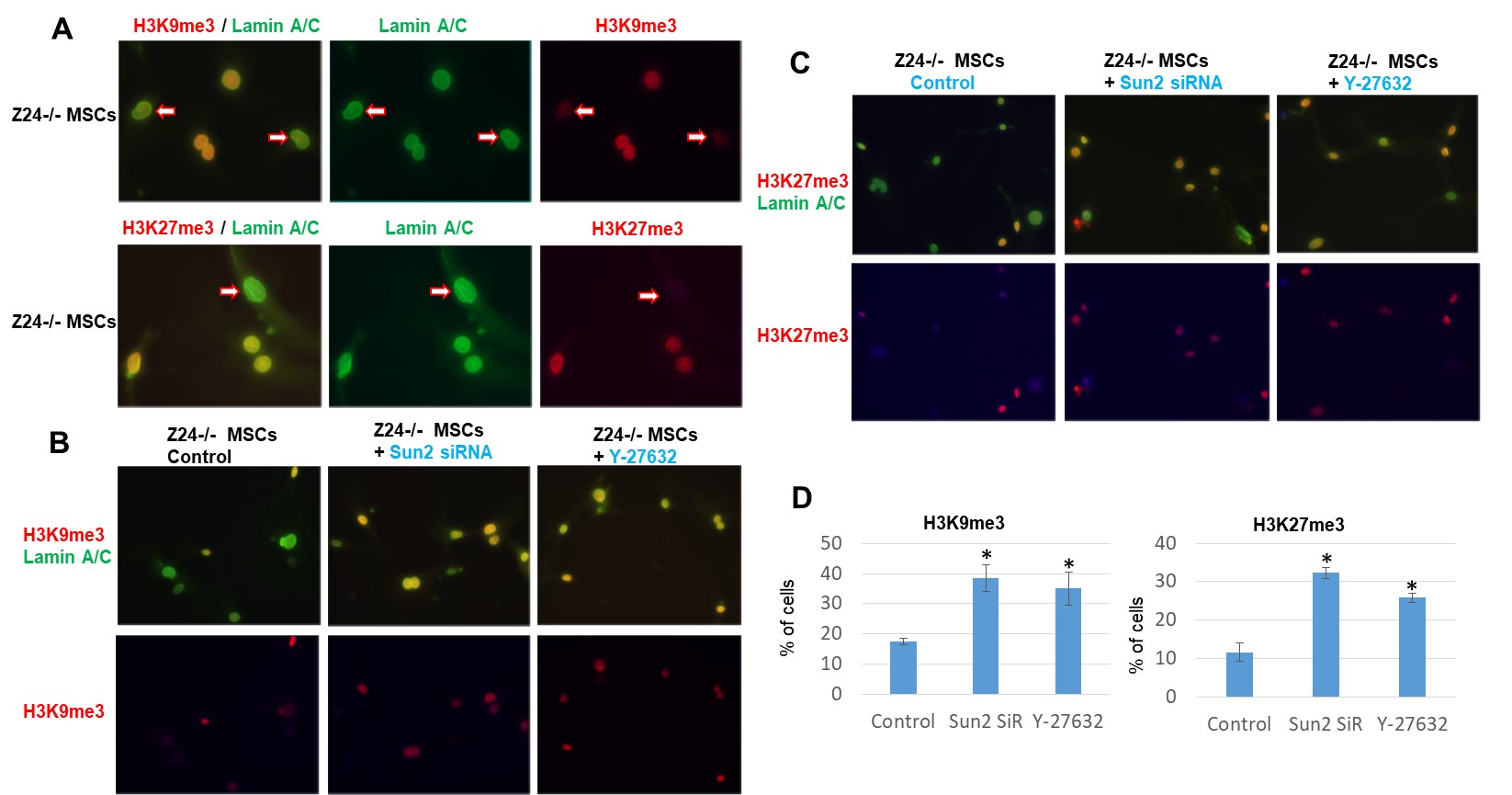
**

**Supplemental Figure 15. RhoA or Sun2 inhibition is effective in restoring epigenetic abnormalities in histone modification in *Z24*^-/-^ MSCs.**

**A.** Immunostaining analysis of the heterochromatin marker H3K9me3 and H3K27me3 in *Z24^-/-^* MSCs. Arrows: nucleus with lower heterochromatin marker and higher level of nuclear blebbing. **B.** Immunostaining analysis of H3K9me3 in *Z24^-/-^* MSCs and *Z24^-/-^* MSCs treated with Sun2 SiRNA or Y-27632. **C**. Immunostaining analysis of H3K27me3 in *Z24^-/-^* MSCs and *Z24^-/-^* MSCs treated with Sun2 SiRNA or Y-27632. **D**. Quantitation of H3K9m33 and H3K27me3 is shown. N>=6. “*” at bar charts indicates p<0.05.

**
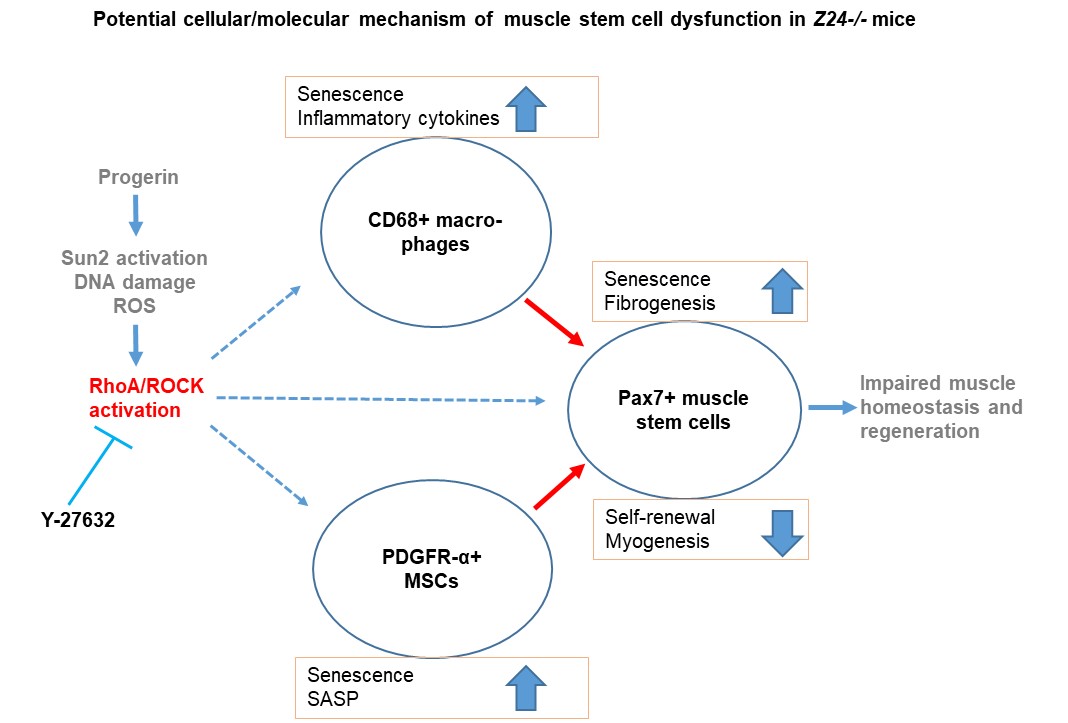
**

**Supplemental Figure 16. A potential mechanism of muscle stem cell dysfunction in *Z24^-/-^* mice.**

Excessive progerin accumulation in nuclear lamina of progeria cells leads to increased nuclear stiffness, Sun2 activation, nuclear blebbing and other senescent phenotypes, which is usually coupled with higher level of DNA damage and ROS production. RhoA activity in PDGFR-α+ MCSs, CD68+ macrophages, and Pax7+ MPCs can all be promoted by Sun2 activation, DNA damage, or ROS in these cells. Accelerated senescence of MSCs or inflammatory cells can cause elevated secretion of SASP factors or pro-inflammatory factors, which can have deleterious paracrine effect on muscle stem cells, and impair their proper functions.

**
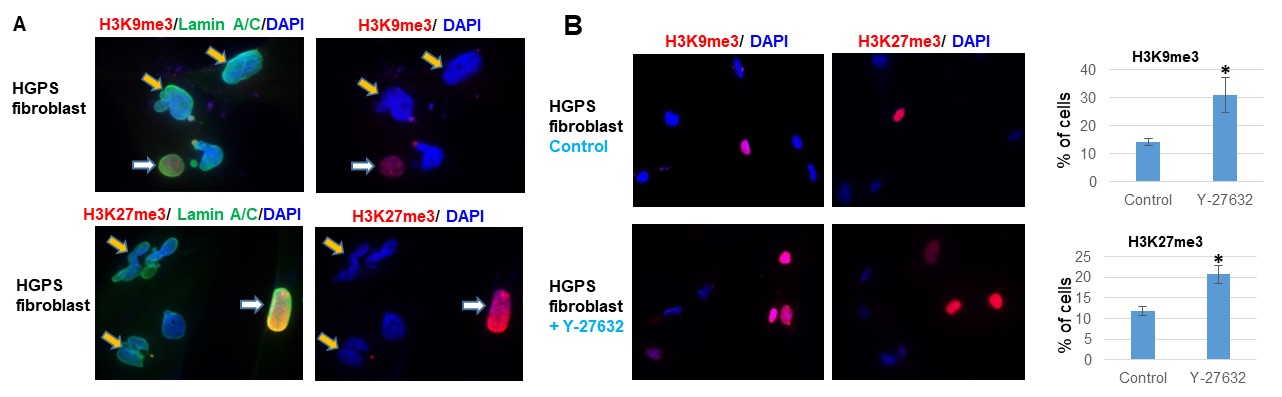
**

**Supplemental Figure 17. RhoA inhibition is effective in restoring epigenetic abnormalities in histone modification in HGPS fibroblasts.**

**A.** Immunostaining analysis of H3K9me3 and H3K27me3 (heterochromatin markers) in HGPS fibroblasts. Orange arrows indicate nuclei with blebbing (lower level of heterochromatin markers); white arrows indicate nuclei without blebbing (higher level of heterochromatin markers). B. Immunostaining analysis of H3K9me3 and H3K27me3) in HGPS fibroblasts treated with Y-27632. Quantitation of H3K9me3 and H3K27me3 in HGPS fibroblasts is shown. N>=6. “*” at bar charts indicates p<0.05.

**
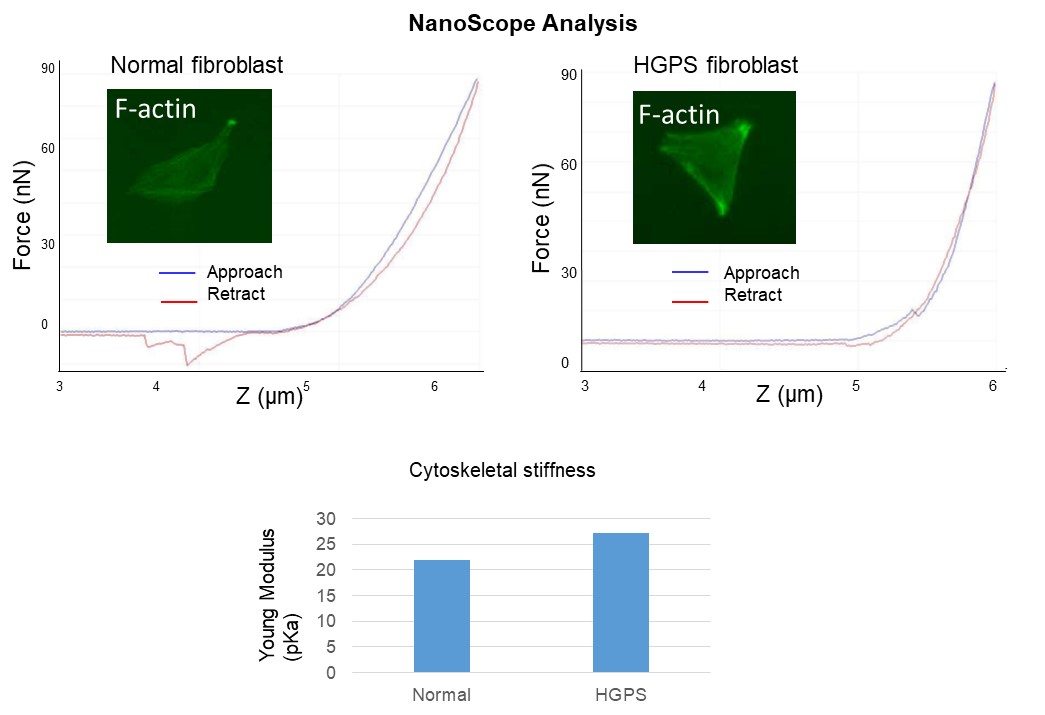
**

**Supplemental Figure 18. HGPS fibroblasts feature higher cytoskeletal stiffness than normal fibroblasts.**

The cytoskeletal stiffness in normal fibroblast and HGPS fibroblasts was tested with the AFM system and NanoScope analysis. N=2.

**Supplemental Table 1: RT-PCR Primer sequences**

| **Gene** | **Primer sequence** |
| --- | --- |
| GAPDH | Forward: TCCATGACAACTTTGGCATTG  Reverse: TCACGCCACAGCTTTCCA |
| TGF-beta1 | Forward: CTCCCGTGGCTTCTAGTGC  Reverse: GCCTTAGTTTGGACAGGATCTG |
| TNF-α | Forward: CCTGTAGCCCACGTCGTAG  Reverse: GGGAGTAGACAAGGTACAACCC |
| TNFR1 | Forward: CCGGGAGAAGAGGGATAGCTT  Reverse: TCGGACAGTCACTCACCAAGT |
| CXCL1 | Forward: CTGGGATTCACCTCAAGAACATC  Reverse: CAGGGTCAAGGCAAGCCTC |
| IL-1alpha | Forward: TCTCAGATTCACAACTGTTCGTG  Reverse: AGAAAATGAGGTCGGTCTCACTA |
| IL-1beta | Forward: GCAACTGTTCCTGAACTCAACT  Reverse: ATCTTTTGGGGTCCGTCAACT |
| IL-6 | Forward: CTGCAAGAGACTTCCATCCAG  Reverse: AGTGGTATAGACAGGTCTGTTGG |
| IL-10 | Forward: ATTTGAATTCCCTGGGTGAGAAG  Reverse: CACAGGGGAGAAATCGATGACA |
| Klotho | Forward: ACTACGTTCAAGTGGACACTACT  Reverse: GATGGCAGAGAAATCAACACAGT |
| MCP1 | Forward: TAAAAACCTGGATCGGAACCAAA  Reverse: GCATTAGCTTCAGATTTACGGGT |
| PDGFR-alpha | Forward: TCCATGCTAGACTCAGAAGTCA  Reverse: TCCCGGTGGACACAATTTTTC |
| PDGFR-beta | Forward: CAAGAAGCGGCCATGAATCAG  Reverse: CGGCCCTAGTGAGTTGTTGT |
| p16 | Forward: AACTCTTTCGGTCGTACCCC  Reverse: GCGTGCTTGAGCTGAAGCTA |
| p21 | Forward: CCTGGTGATGTCCGACCTG  Reverse: CCATGAGCGCATCGCAATC |
| Collagen I | Forward: GCTCCTCTTAGGGGCCACT  Reverse: CCACGTCTCACCATTGGGG |
| mTORC1 | Forward: CACCAGAATTGGCAGATTTGC  Reverse: CTTGGACGCCATTTCCATGAC |
| Sun1 | Forward: CCAGGCTATTGATTCGCACAT  Reverse: GTGGTTGATACTAAAAGCTGGCT |
| Sun2 | Forward: ACTCTCAGGATGATAACGATGGC  Reverse: AGACTCGCTGTAGTAGGAGGT |

|  |
| --- |
